# Spatiotemporal Ca^2+^ nanodomain remodeling at MERCS regulates mitochondrial proteostasis

**DOI:** 10.1093/procel/pwaf109

**Published:** 2025-12-08

**Authors:** Yanan Lv, Xuejing Zhao, Di Li, Zhaoqi Hao, Yue Zhao, Yuhang Zhou, Yujing Zhang, Han Chen, Zhongbing Lu, Dong Li, Yuting Guo

**Affiliations:** College of Life Sciences, University of Chinese Academy of Sciences, Beijing 100049, China; College of Life Sciences, University of Chinese Academy of Sciences, Beijing 100049, China; Beijing National Laboratory for Condensed Matter Physics, Institute of Physics, Chinese Academy of Sciences, Beijing 100190, China; University of Chinese Academy of Sciences, Beijing 100049, China; Liyang Tianmu Lake Center of Applied Medical Physics Co., Ltd, Changzhou 213333, China; College of Life Sciences, University of Chinese Academy of Sciences, Beijing 100049, China; College of Life Sciences, University of Chinese Academy of Sciences, Beijing 100049, China; College of Life Sciences, University of Chinese Academy of Sciences, Beijing 100049, China; College of Life Sciences, University of Chinese Academy of Sciences, Beijing 100049, China; College of Life Sciences, University of Chinese Academy of Sciences, Beijing 100049, China; College of Life Sciences, University of Chinese Academy of Sciences, Beijing 100049, China; School of Life Sciences, Tsinghua University, Beijing 100084, China; College of Life Sciences, University of Chinese Academy of Sciences, Beijing 100049, China

**Keywords:** calcium transients, mitochondrial stress response, Mito–ER interaction, Alzheimer’s disease, super-resolution microscopy

## Abstract

Mitochondrial calcium fluxes serve as pivotal regulators of optimal organellar function and cellular viability, yet the spatiotemporal regulation of nanodomain Ca^2+^ transients at mitochondria–ER contact sites (MERCS) and their integration into adaptive mitochondrial stress signaling remain unresolved. In this study, we employed custom-built high temporal-spatial resolution GI/3D-SIM imaging techniques to achieve nanoscale resolution of calcium transients. We identify that MERCS-localized calcium oscillations gate retrograde stress signaling. Mechanistically, we demonstrate that augmented mitochondria-associated ER membrane (MAMs) connectivity unexpectedly attenuated global mitochondrial Ca^2+^ efflux, which triggering ATF5 shuttling-mediated transcriptional licensing and calcium-sensitive epigenetic reprogramming that synergistically activating stress-resilience programs. Quantitative protein expression and transcriptome analyses confirm that CsA-mediated calcium retention mimics MAMs induction preserves mitochondrial integrity and protecting cells from apoptosis in Aβ1-42-challenged neurons through synchronized UPR^mt^ activation. Our findings reveal a novel mechanism by which MERCS decode proteotoxic stress into transcriptional and epigenetic adaptations, offering therapeutic potential for neurodegenerative diseases.

## Introduction

Calcium ions serve as ubiquitous second messengers that orchestrate diverse cellular processes across subcellular compartments, including the endoplasmic reticulum (ER), mitochondria, and cytosol (Benedetti et al., 2025; Zheng et al., 2022). In mitochondria, calcium homeostasis plays a dual role in both sustaining bioenergetic signaling and determining cell survival, with its dysregulation being mechanistically linked to multiple pathologies including Parkinson’s disease (PD) (Matuz-Mares et al., 2022), type 2 diabetes mellitus (T2DM) (Liu et al., 2019), and Alzheimer’s disease (AD) (Jadiya et al., 2019; Melber and Haynes, 2018; Sorrentino et al., 2017; Valasani Koteswara Rao et al., 2014; Zampese et al., 2011, Zhang et al., 2023). Mitochondrial calcium influx occurs primarily at membrane contact sites (MCSs), where ER-resident inositol 1,4,5-trisphosphate receptors (IP3Rs) and ryanodine receptors (RyRs) interface with mitochondrial voltage-dependent anion channels (VDACs) through molecular tethers like glucose-regulated protein 75 (GRP75, encoded by *HSPA9*) (Giorgi et al., 2018). The mitochondria-associated ER membrane (MAM) forms a nanoscale calcium exchange platform, with efflux mediated by the mitochondrial Na^+^/Ca^2+^ exchanger (NCLX, encoded by *SLC8B1*) and permeability transition pore (mPTP) (Shoshan-Barmatz and De, 2017; Yamamoto, 2021). Pathological MAM hyperconnectivity drives mitochondrial Ca^2+^ overload (>500 nmol/L matrix concentration), inducing mPTP-driven cytochrome c release and apoptosis—a hallmark of neurodegenerative cascades (Bernardi et al., 2023; Jadiya et al., 2019; Valasani Koteswara Rao et al., 2014). Despite progress in mapping static MAM architectures, the *in vivo* spatiotemporal regulation of calcium nanodomains (<90 nm) at MERCS and their functional interplay with mPTP gating kinetics remain enigmatic (Cardenas et al., 2010; Dong et al., 2024; Kornmann et al., 2009; Lopez-Crisosto et al., 2021).

Mitochondrial proteostasis-governing ATP synthesis, lipid metabolism, and Ca^2+^ buffering-deteriorates during aging and neurodegeneration (Eisner et al., 2018). To counteract proteotoxic stress, evolutionarily conserved quality control pathways, including mitochondrial dynamics, mitophagy, and the mitochondrial unfolded protein response (UPR^mt^), are activated. The UPR^mt^ is an evolutionarily conserved mitochondrial stress response pathway that can be induced in *Caenorhabditis elegans* (*C. elegans*), *Drosophila*, and *Homo sapiens* (Martinus et al., 1996). The UPR^mt^, operational from *C. elegans* to humans, restores proteostasis via induction of chaperones (HSP60, CLPP) and proteases (LONP1) (Cheng et al., 2013; Shpilka and Haynes, 2018). Its activation requires multi-tiered regulation: (i) transcriptional control by *ATF5* (mammalian ortholog of *ATFS-1*) (Fiorese et al., 2016); (ii) epigenetic modulation via jumonji/HDA-1 histone modifiers and DVE-1/SATB2 (Nargund et al., 2012; Shao et al., 2020; Wang et al., 2022); and (iii) post-translational tuning of DVE-1/SATB2 chromatin remodelers (Melber and Haynes, 2018; Shao et al., 2020). These findings highlight the necessity for coordinated multilevel inputs to precisely calibrate UPR^mt^ activation in accordance with pathophysiological demands. However, how organelle-derived signals—particularly MAM-mediated Ca^2+^ effluxes—interface with these nuclear programs to calibrate UPR^mt^ activation remains unresolved.

Herein, we delineate a MERCS-nucleus signaling axis that decodes proteotoxic stress through calcium-dependent ATF5 dislocating and chromatin restructuring. Through home-made high temporal-spatial resolution imaging methods, including GI-SIM (Guo et al., 2018), 3D-SIM, and lattice light-sheet microscopy, we demonstrate that the expanded MERCS constrains mitochondrial Ca^2+^ efflux via spatial confinement of mPTP opening. This nanodomain Ca^2+^ retention licenses two convergent nuclear adaptations: (i) ATF5 nuclear translocation activating stress-responsive transcription; (ii) CAMK4-driven H3K27 acetylation inducing chromatin loosen. This transcriptional and epigenomic integration enhances UPR^mt^-related gene expression, which alleviates Aβ1-42-induced neuronal apoptosis. Strikingly, genetic (REDMAP), pharmacological (mPTP inhibition), and optogenetic MAM manipulations all converged on calcium-sensitive 3D genome restructuring to rescue proteostasis. Our work establishes that mitochondrial proteostatic stress is decoded through a MAM-calcium-ATF5/epigenomic remodeling signaling cascade that: (i) gates mPTP permeability via membrane tethering dynamics; (ii) encodes proteotoxic stress into transcriptional (ATF5) and epigenetic (CAMK4) nuclear signals; (iii) therapeutically recalibrates mito-nuclear crosstalk in neurodegenerative cascades.

## Results

### Mitochondrial calcium extrusion is governed by mitochondria–ER contact sites

**Figure 1.**
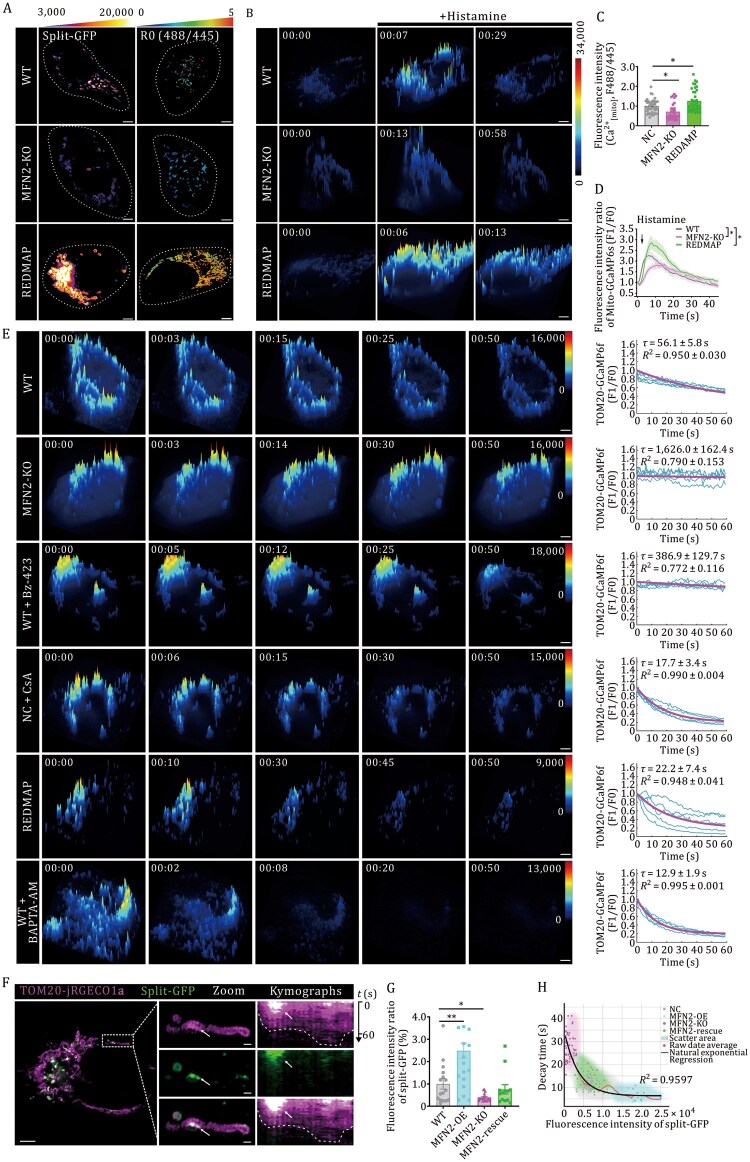
**Mito-ER interaction dynamics regulate compartmentalized calcium signaling**. (A) Representative GI-SIM imaging of Mito-ER interaction density on the left using the Split-GFP reconstitution system. Left: Representative maximum-intensity projections of control versus MFN2-KO/REDMAP cells, with contact sites pseudo-colored by fluorescence intensity ratio (scale: blue [low] to yellow [high]). Right: Corresponding ratiometric images (mito-CFP-GCaMP6s) of mitochondrial calcium dynamics. Scale bars: 5 µm. (B) 3D surface plots of summed ${\text{Ca}}_{\left[\text{mito}\right]}^{2+}$ transients detected by GI-SIM in WT, MFN2*-*KO and REDMAP cells transfected with mito-GCaMP6s. Scale bars, 5 µm. (C) Statistical analysis of the fluorescence intensity ratio of mito-CFP-GCaMP6s. Comparison of means (±SEM) in REDMAP, MFN2-KO, and WT cells. **P < 0.01 (two-tailed unpaired Student’s *t*-test). (D) Representative fluorescent traces of periodic mitochondrial Ca^2+^ transients recorded by mito-GCaMP6s induced by histamine (100 µmol/L) in WT, MFN2-KO, and REDMAP cells. Comparison of means (±SEM). *P < 0.05 (two-tailed unpaired Student’s *t*-test). (E) 3D time-series surface plots of summed Ca^2+^ transients detected by GI-SIM in the groups as indicated. Representative fluorescent traces of periodic mitochondrial Ca^2+^ transients recorded by TOM20-GCaMP6f from five randomly selected cells across three independent experiments are shown as blue curves, and exponential fitting of the fluorescence decay is shown in magenta. Scale bars, 5 µm. (F) Kymographic analysis of the GI-SIM image in the white dashed box. Scale bars, 1 µm (spatial), 60 s (temporal). (G) Quantitative Mito-ER contact intensity across genetic models: wild-type (WT), MFN2-KO, MFN2-OE, and MFN2-rescue cells. Statistical significance was determined by two-tailed unpaired Student’s *t*-test (*P < 0.05, **P < 0.01 vs. WT). (H) Quantitative correlative analysis of Mito-ER contact density (Split-GFP fluorescence intensity) and calcium signal decay kinetics (TOM20-GCaMP6f τ values) across genetic perturbation groups. See also Fig. S1.

While mitochondrial calcium influx at MCSs is well-characterized, the spatiotemporal regulation of calcium efflux at organelle contact sites remains poorly understood. To probe the relationship between mitochondrial calcium transients and MERCS, we employed both loss-of-function (MFN2-KO, GRP75-KD) and gain-of-function (REDMAP-engineered) (Zhou et al., 2022a) cellular models that respectively disrupt and enhance MERCS. Using Split-GFP-based MAM mapping coupled with ratiometric mito-CFP-GCaMP6s imaging, we confirmed that MFN2-KO and GRP75-KD cells exhibited both disrupted MAM integrity (Figs. 1A, left and S1A–D) and significantly lower mitochondrial calcium levels compared to wild-type (WT) controls (Fig. 1A and 1C). Conversely, cells with REDMAP-enhanced MERCS showed elevated mitochondrial calcium retention (Fig. S1E). Consistent with these findings, real-time monitoring of calcium flux during histamine-induced ER Ca^2+^ release revealed severely compromised mitochondrial calcium import in MFN2-KO cells (Fig. 1B and 1D), while REDMAP-enhanced MERCS displayed enhanced calcium import. These genetic models thus establish tools for studying MAM-deficient or MAM-enhanced conditions.

We next investigated whether mitochondrial calcium efflux dynamics depend on MERCS integrity using live-cell imaging with TOM20-GCaMP6f, a cytosolic-facing genetically encoded calcium indicator specifically targeted to mitochondrial-cytosolic interface nanodomains (Fig. S1B and S1F–H). Systematic quantification of calcium flux decay kinetics (via time constant τ) revealed prolonged calcium retention in MFN2-KO cells (τ = 1,626 ± 162.4 s, *n *= 25) and GRP75-KD cells (τ = 562.3 ± 102.2 s, *n *= 12; Fig. S1I) compared to WT (τ = 56.1 ± 5.8 s, *n *= 43), mirroring Bz-423-treated cells (τ = 386.9 ± 129.7 s, *n *= 12). Conversely, REDMAP-engineered cells with reinforced MAMs showed accelerated signal attenuation (τ = 22.2 ± 7.4 s, *n *= 23), comparable to pharmacological inhibition of calcium efflux via cyclosporin A (CsA: 5 µmol/L for 2 h; τ = 17.7 ± 3.4 s, *n *= 18) or BAPTA-AM (a highly selective chelator of intracellular calcium ions, 50 µmol/L for 15 min; τ = 12.9 ± 1.9 s, *n *= 16, Fig. 1E; Video S1) (Crompton et al., 1988; Karch and Molkentin, 2014). Neither CGP37157 (mitochondrial NCLX inhibitor, 10 µmol/L for 2 h) nor DRP1-OE (to induce mitochondrial fragmentation, Fig. S1I) significantly altered efflux kinetics. Notably, mito-GCaMP6s recordings indicated that reduced mitochondrial outer surface calcium flow in MAM-deficient cells was independent of calcium influx. These results collectively suggest that mitochondrial calcium extrusion at MAMs is primarily regulated by physical interorganellar tethering rather than classical transport mechanisms.

To achieve nanoscale resolution of calcium flux regulation at mitochondria–ER contact sites, we performed GI-SIM imaging at 2 s/frame in combination with a Split-GFP proximity labeling system, in which GFP reconstitution generates a distance-dependent fluorescent signal. Live-cell dual-channel imaging of cells co-expressing TOM20-jRGECO1a (magenta) displayed compartmentalized calcium dynamics, with accelerated signal decay kinetics at GFP-reconstituted MERCS microdomains (white arrow, Fig. 1F, Video S2) compared to distal mitochondrial subregions within the same mitochondrion, revealing nanoscale functional polarization within individual mitochondria. Quantitative MERCS profiling demonstrated that MFN2 ablation reduced contact density by 62.3% ± 5.1% compared to WT, rescued by *MFN2* re-expression, and a 262.5% ± 8.1% increase in MFN2 overexpression (MFN2-OE) cells (Fig. 1G). Correlative analysis across genetic perturbations demonstrated a strong inverse correlation between MERCS density (Split-GFP intensity) and calcium decay kinetics (TOM20-GCaMP6f τ values): WT (6,688.0 ± 3,056.2 intensity/µm^2^, τ = 12.6 ± 5.5 s), MFN2-KO (1,369.9 ± 910 intensity/µm^2^, τ = 27.3 ± 5.9 s), and MFN2-OE (20,277.8 ± 4,411.5 intensity/µm^2^, τ = 6.3 ± 1.9 s) (Fig. 1H, *R*^2^ = 0.9597, *P *< 0.0001 by natural exponential regression). This quantitative framework establishes MERCS tethering density as a geometric determinant of calcium efflux efficiency, mechanistically linking membrane contact topology to compartmentalized calcium extrusion.

### Mito-ER interactions modulate mitochondrial proteostasis

Emerging evidence implicates that the Mito-ER interactions are closely linked to a wide spectrum of metabolic diseases, such as PD (Liu et al., 2019) and AD (Zampese et al., 2011; Zhang et al., 2023), with UPR^mt^ activation contributing to the treatment of aging-related diseases (Melber and Haynes, 2018; Sorrentino et al., 2017). While Mito-ER interactions are proposed to regulate mitochondrial stress pathways through metabolic modulation (Dong et al., 2024; Lopez-Crisosto et al., 2021), their mechanistic role in modulating proteostatic insults remains unclear.

**Figure 2.**
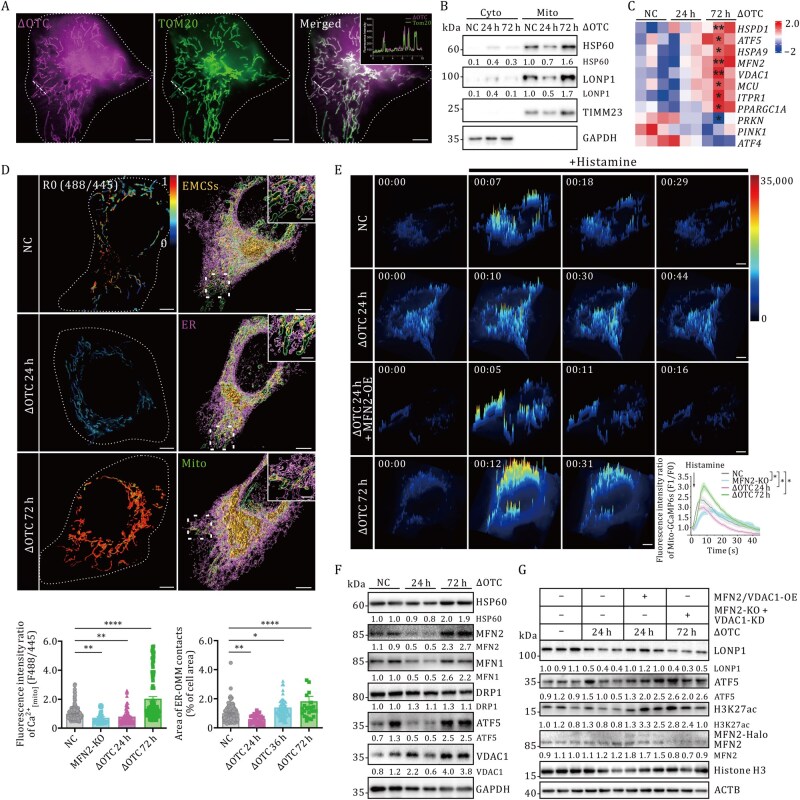
**MAMs are essential for UPR^mt^ initiation**. (A) GI-SIM images showing colocalization of ΔOTC-mCherry with mitochondrial membrane marker TOM20-mEmerald. The fluorescence intensity profiles of unfolded protein (magenta) and mitochondria (green) corresponding to the white dashed line are indicated. (B) Quantification of UPR^mt^ marker protein expression in isolated mitochondria from cells transfected with NC, ΔOTC for short-term (24 h) and long-term (72 h). (C) The expression of MAM-related genes is shown in the clustered heatmap. The color scale indicates the level of log_2_ fold change (blue, low; red, high). (D) Left column: Pseudocolor ratiometric images (F488/F445) of mitochondrial calcium dynamics monitored by mito-CFP-GCaMP6s. Scale bars, 5 µm (main images), 1 µm (insets). Right column: 3D-SIM imaging depicting mitochondria (green) and ER (magenta) interactions in control versus ΔOTC-transfected cells. Mito-ER interaction areas are highlighted in yellow using the Imaris Surfaces tool. Magnified views of the dashed box areas are shown in the upper right corner of the respective 3D images. (E) 3D surface plots of summed ${\text{Ca}}_{\left[\text{mito}\right]}^{2+}$ transients detected by GI-SIM in U2OS cells co-expressing mito-GCaMP6s and mCherry-ΔOTC for varied times. Representative fluorescent traces of periodic mitochondrial Ca^2+^ transients recorded by mito-GCaMP6s induced by histamine (100 µmol/L) in control, MFN2-KO, and ΔOTC transfected cells are indicated. All data are expressed as means (±SEM) from individual cells. Scale bars, 5 µm. Statistical significance was determined by two-tailed unpaired Student’s *t*-test (*P < 0.05, **P < 0.01, ****P < 0.0001 vs. untreated controls). (F and G) Western blot analysis of UPR^mt^ marker protein levels. Protein levels were normalized against ACTB, GAPDH, or Histone H3 and set to 1.00 in the corresponding control cells. See also Fig. S2.

To address this, we constructed a mitochondrial proteostatic stress model by overexpressing a truncated ornithine transcarbamylase (ΔOTC-mCherry) lacking critical folding domains in the mitochondrial matrix (Zhao et al., 2002). Live imaging confirmed mitochondrial-specific ΔOTC-mCherry localization (Fig. 2A). Temporal analysis of UPR^mt^ markers revealed biphasic regulation: cytoplasmic and mitochondrial fractions demonstrated transient suppression of HSP60 and LONP1 protein levels at 24 h post-transfection (ΔOTC 24 h), followed by sustained UPR^mt^ activation at 72 h (ΔOTC 72 h; Figs. 2B and S2J). Transcriptomic and protein analyses during activation showed selective upregulation of Mito-ER tethers (MFN1: 2.5-fold, MFN2: 3.0-fold, VDAC1: 3.2-fold; *P *< 0.01 vs. control) without affecting mitophagy markers (*PINK1*/*PRKN*) and UPRer effectors (*ATF4*, BIP; Figs. 2C, S2A and S2B, Table S2).

To extend these protein quantification results, we performed high-resolution imaging analysis, and the results demonstrated Mito-ER interface remodeling: 72 h ΔOTC increased contact frequency (1.8-fold, *P *< 0.01) and surface area (2.3-fold, *P *< 0.001), while 24 h treatment reduced both parameters (yellow, Fig. 2D; Video S3). Consistent with this finding, we find mitochondrial fission slightly elevated in the 72 h ΔOTC group compared to WT, and mitochondrial length has a trend of shortening (Fig. S2C and S2D). In contrast, mitochondrial counts (Fig. S2E) and mitochondrial-nuclear interactions (Fig. S2F) remained unaltered across conditions. Concordantly, mitochondrial calcium (${\text{Ca}}_{\left[\text{mito}\right]}^{2+}$) displayed temporal dynamics aligned with UPR^mt^ status: suppressed under UPR^mt^-inactive conditions (24 h ΔOTC) and elevated during UPR^mt^ activation (72 h ΔOTC) (Figs. 2D and S2G), without alterations in cytosolic (Fig. S2H) or lysosomal calcium stores (Fig. S2I). Histamine-induced ER Ca^2+^ release showed impaired mitochondrial uptake at 24 h (42% ± 5% reduction, *P *< 0.05) versus enhanced import at 72 h (160% ± 12% increase, *P *< 0.05) (Fig. 2E; Video S4). Genetic manipulation of Mito-ER tethers established causality: MFN2/VDAC1 overexpression rescued UPR^mt^ suppression at 24 h (2.8-fold recover vs. 24 h ΔOTC; *P *< 0.01), while interfering Mito-ER tethering protein (VDAC1/MFN2) attenuated activation at 72 h (HSP60: 73% ± 5%, ATF5: 26% ± 7% inhibition; *P *< 0.05) (Figs. 2G, S2K and S2L). Collectively, these data demonstrate that Mito-ER interactions are critical architectural determinants of UPR^mt^ activation.

### MERCS regulate proteostatic resilience through fine-tuning mitochondrial calcium transients

**Figure 3.**
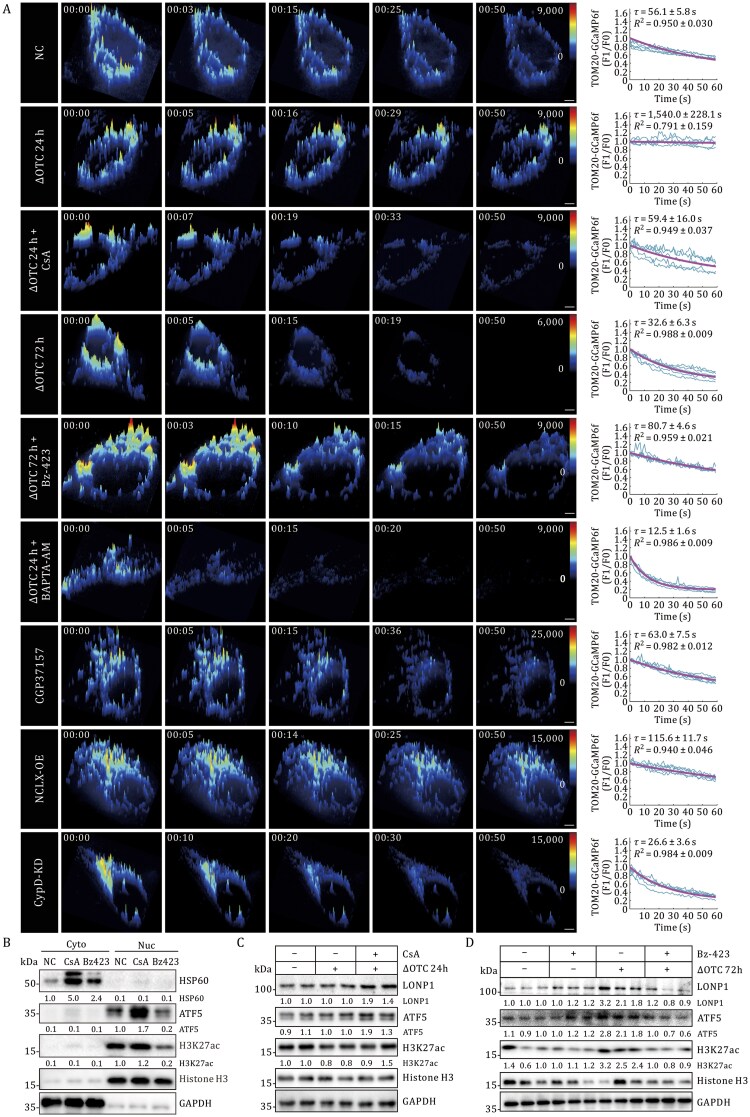
**Hindering calcium transients drives UPR^mt^ activation**. (A) 3D time-series surface plots of summed Ca^2+^ transients detected by GI-SIM in U2OS cells. Representative fluorescent traces of periodic mitochondrial Ca^2+^ transients recorded by TOM20-GCaMP6f from five randomly selected cells in each group are shown as blue curves, and exponential fitting of the fluorescence decay is shown in magenta. Scale bars, 5 µm. (B) Subcellular fractionation immunoblots of ATF5/HSP60 under pharmacological interventions (CsA/Bz-423). Loading controls: GAPDH (cytoplasm), Histone H3 (nucleus). (C and D) Quantification of protein expression levels of UPR^mt^ markers in CsA (C) and Bz-423 (D) treatment groups compared to the untreated group. Protein levels were normalized against ACTB, GAPDH, or Histone H3 and set to 1.00 in the corresponding control cells. See also Fig. S3.

To elucidate the regulatory mechanisms underlying MERCS-mediated proteostatic adaptation, we performed live-cell GI-SIM imaging to monitor MERCS-regulated mitochondrial calcium transients in control and ΔOTC-transfected cells co-expressing TOM20‑GCaMP6f. Potential artifacts in GCaMP6f signal due to intergroup variation were ruled out by assessing Pearson’s correlation, pH, and mitochondrial dynamic movement (Fig. S3A–E). Quantitative analysis of mitochondrial calcium transients revealed distinct kinetic profiles under different UPR^mt^ states. Cells with suppressed UPR^mt^ (24h ΔOTC) showed prolonged calcium efflux (τ = 1,540 ± 228.1 s, *n *= 17) compared to normal controls (τ = 56.1 ± 5.8 s, *n *= 43), resembling the phenotypes observed in MFN2-KO (τ = 1,626 ± 162.4 s, *n *= 25) and NCLX-OE (*SLC8B1* overexpression) cells (τ = 115.6 ± 11.7 s, *n *= 12; Figs. 3A and S3C). This prolonged efflux was reversed by CsA (mPTP inhibitor; τ = 59.4 ± 16 s, *n *= 18). In contrast, UPR^mt^-activated cells (72 h ΔOTC) exhibited accelerated calcium clearance (τ = 33 ± 6 s, *P *< 0.01, *n *= 23), similar to CypD-KD cells (Fig. S3G). This accelerated kinetics was abolished by Bz-423, an mPTP opening inducer (τ = 80.7 ± 4.6 s, *n *= 16; Fig. 3A; Video S5). These results implied that mitochondrial calcium extrusion kinetics—modulated by Mito-ER interactions—act as a rheostatic node governing mitochondrial stress response activation.

Integrated transcriptomic and proteomic analyses confirmed that attenuated mitochondrial calcium efflux triggers UPR^mt^ activation. Treatment with CsA or BAPTA-AM restored UPR^mt^ markers—including ATF5, HSP60, LONP1, and H3K27ac—in both control and 24 h ΔOTC-expressing cells (Figs. 3B–D, S3H, S3I, and S3M). Conversely, promoting mitochondrial calcium extrusion via Bz-423 (20 µmol/L) significantly suppressed ATF5 and LONP1 induction in 72 h ΔOTC cells (60%–72% reduction; Figs. 3D and S3N). Similarly, NCLX-OE led to a moderate increase in Ca^2+^ efflux and mild attenuation of UPR^mt^ activation (Fig. S3F and S3J). In contrast, neither NCLX-KD (Fig. S3K) nor Ru360-mediated MCU inhibition (Fig. S3L) notably altered UPR^mt^-related protein levels. Together, these data demonstrate that UPR^mt^ activation depends principally on mitochondrial calcium transient retention, which is primarily regulated by mPTP opening and can be facilitated by either enhanced mitochondria–ER contacts or pharmacological inhibition of calcium efflux. Our findings uncover a calcium efflux–gated regulatory paradigm governing mitochondrial proteostatic adaptation.

### Mitochondrial calcium dynamics mediate UPR^mt^ activation through coordinated ATF5 trafficking and chromatin reorganization

**Figure 4.**
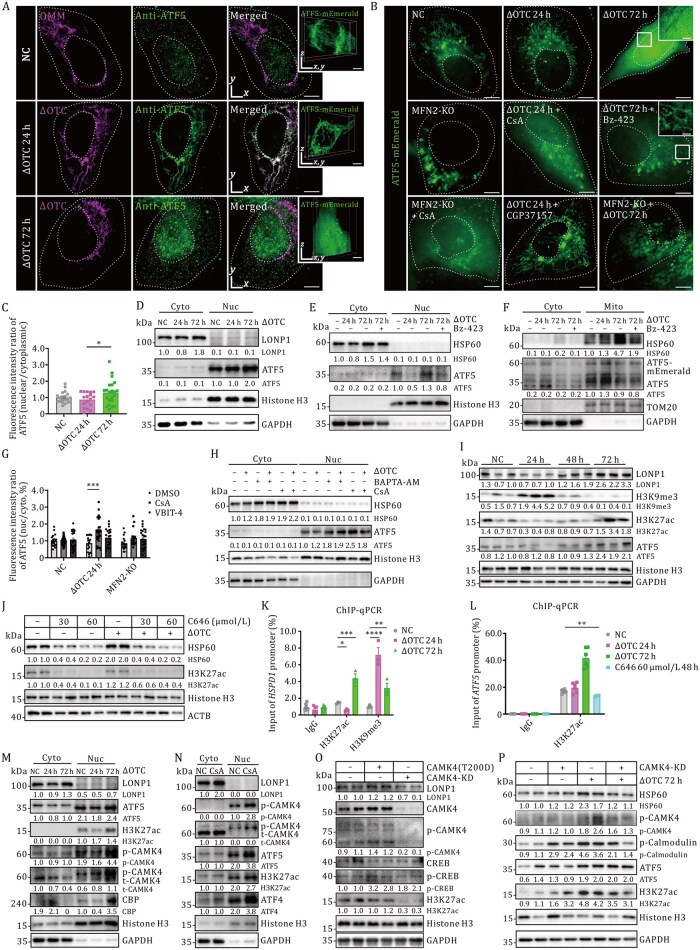
**Mitochondrial-nuclear communication via ATF5 trafficking and chromatin remodeling orchestrates calcium-dependent UPR^mt^ activation**. (A) Representative GI-SIM imaging of endogenous ATF5 sublocation in control versus mitochondrial stress conditions (ΔOTC 24 h/72 h). 3D Lattice light-sheet images of ATF5-mEmerald from the corresponding transfection groups are presented. Scale bars, 5 µm in GI-SIM and Lattice light-sheet images. (B) Subcellular redistribution of ATF5-mEmerald in WT versus MFN2-KO cells under pharmacological perturbations (ΔOTC ± CsA [5 µmol/L], Bz-423 [20 µmol/L], CGP37157 [10 µmol/L]). Scale bars, 5 µm (main images); 1 µm (insets). (C) Quantification of ATF5 nuclear/cytoplasmic (N/C) fluorescence ratios across ΔOTC transfection durations (0–72 h). Data represent mean ± SEM. *P < 0.05 vs. control (One-way ANOVA). (D–F) Subcellular fractionation immunoblots of ATF5 under temporal mitochondrial stress (ΔOTC 24 h and 72 h) and pharmacological interventions (Bz-423). Loading controls: GAPDH (cytoplasm), Histone H3 (nucleus). (G) Pharmacological modulation of ATF5 N/C ratios in ΔOTC-transfected cells. ***P < 0.001 vs. NC (Two-way ANOVA). (H) Subcellular fractionation immunoblots of ATF5 under BAPTA or CsA treatment. Loading controls: GAPDH (cytoplasm), Histone H3 (nucleus). (I) Immunoblot of H3K9me3 and H3K27ac expression in ΔOTC transfection time gradient. Anti-LONP1 and Anti-ATF5 serve as markers for UPR^mt^ suppression (lanes 4–6) or activation (lanes 10–12). (J) Immunoblot of UPR^mt^ markers expression under C646-mediated histone acetylation inhibition. (K and L) ChIP-qPCR analysis monitoring H3K27ac and H3K9me3 enrichment levels at the gene regulatory region of *HSPD1* (K) and *ATF5* (L). Statistical analysis was normalized against the input sample according to the percent input method across three independent experiments (*n *= 3). Two-way ANOVA was used to compare ΔOTC 24 h and ΔOTC 72 h with the untreated group (*P < 0.05, **P < 0.01, ***P < 0.001, ****P < 0.0001). (M and N) Western blot analysis of H3K27ac, active CAMK4 protein (p-CAMK4^Thr196/Thr200^) expressions using N/C fractions. (O and P) Western blot analysis of HSP60/LONP1, H3K27ac expression levels in gain- and loss-of-function of CAMK4 compared with control cells. See also Fig. S4.

To delineate the mechanistic link between mitochondrial calcium transients and UPR^mt^ activation, we systematically characterized calcium-mediated transcriptional-epigenetic reprogramming across UPR^mt^ modulation paradigms. GI-SIM and Lattice light-sheet microscopy (Chen et al., 2014) imaging demonstrated subcellular redistribution of ATF5 (transcription factor of UPR^mt^), with mitochondrial sequestration in UPR^mt^-suppressed cells (24 h ΔOTC) versus nuclear accumulation in UPR^mt^-activated cells (72 h ΔOTC, Fig. 4A and 4C). Nucleocytoplasmic fractionation confirmed stress-induced dislocating ATF5 to nuclear (Figs. 4D and S4B). To amplify the fluorescence signal of ATF5 expression, we overexpressed ATF5-mEmerald across experimental groups. In control and MFN2-KO cells, ATF5-mEmerald was predominantly mitochondrial targeting (Fig. 4B). In contrast, prolonged mitochondrial stress triggered its clear accumulation in the cytoplasm and nucleus (Fig. 4B and 4D), a pattern also observed in cells with compromised mitochondrial import (Fig. S4A). *MFN2* depletion attenuated this redistribution, and the effect was partially reversed by Bz-423 treatment (Fig. 4B, 4E, and 4F). Complementary pharmacological studies demonstrated that either CsA or BAPTA restored UPR^mt^ activation in MFN2-KO and short-term ΔOTC models, concurrently promoting ATF5 nuclear entry (Figs. 4B, 4G, 4H and S4J). VBIT-4, however, had no significant effect (Fig. 4G). Together, these results establish that attenuated mitochondrial calcium transients direct calcium-mediated nuclear shuttling of ATF5 to initiate the UPR^mt^.

Additionally, we investigate the chromatin remodeling involved in calcium-governed UPR^mt^ activation. Super-resolution imaging uncovered biphasic nuclear reorganization during UPR^mt^ progression: early chromatin condensation (24 h) transitioned to decompaction at later stages (72 h), temporally aligned with UPR^mt^ markers (Fig. S4C). 3D-SIM imaging of LaminB1 revealed dynamic nucleoplasmic reticulum (NR) remodeling, with NR density peaking at 24 h before declining to basal levels by 72 h (Fig. S4C), implicating epigenomic modification in UPR^mt^ regulation. Consistent with these results, global H3K9me3 (heterochromatin marker) significantly decreased while H3K27ac (euchromatin mark) increased 2.3-fold (*P *< 0.01) during prolonged stress, paralleling ATF5 upregulation (Figs. 4I and S4K). Inhibition of histone acetylation with C646 attenuated HSP60 induction (Figs. 4J, S4D, S4E, and S4L), indicating that acetylation-dependent chromatin accessibility modulates UPR^mt^ activation. ChIP-qPCR and ATAC-seq analyses confirmed that the *HSPD1* promoter was enriched with H3K27ac (4.8-fold at 72 h) (Figs. 4K and S4I), an effect abolished by C646 treatment (Figs. 4L and S4L; Table S1).

To further delineate this epigenetic axis, we performed RNA-seq and ATAC-seq under conditions of mitochondrial calcium perturbation (PRJEB102168). The results revealed upregulation of calcium-sensitive epigenetic regulators *CAMKK* and *CAMK4* (2.1- and 2.4-fold induction) following 72 h ΔOTC transfection (Fig. S4F; Table S2) or CsA treatment (Fig. S4G; Table S3). Subcellular fractionation analysis confirmed nuclear accumulation of active CAMK4 (phosphor-CAMK4^Thr196/Thr200^: 2.0 ± 0.3-fold, *P *< 0.01; CBP: 3.2 ± 0.4-fold, *P *< 0.001) in CsA and 72 h ΔOTC-prompted UPR^mt^ models, accompanied by elevated H3K27ac levels (Figs. 4M, 4N, S4M and S4N). ChIP-qPCR verified p-CREB enrichment at the *HSPD1* promoter, a known substrate of CAMK4 (Fig. S4H). Gain- and loss-of-function studies using constitutively active CAMK4 (T200D) mutant and siRNA-mediated CAMK4-KD (75% efficiency, *P *< 0.001) respectively enhanced and abolished 72 h ΔOTC-induced UPR^mt^ activation (Figs. 4O, 4P and S4O), establishing CAMK4 as a critical mitochondrial calcium-sensitive mediator linking mitochondrial stress to phase-specific chromatin remodeling.

Together, our results reveal a calcium efflux-dependent bifunctional regulatory axis that orchestrates mitochondrial proteostasis through dual-layered control of (1) stress-responsive transcriptional programs mediated by ATF5, and (2) chromatin topological remodeling via the CAMK4-CREB-H3K27ac pathway, thereby mechanistically coupling ionic flux to organellar proteostatic adaptation.

### UPR^mt^-driven proteostatic adaptation licenses cell integrity through spatiotemporal remodeling of mitochondrial calcium transients

**Figure 5.**
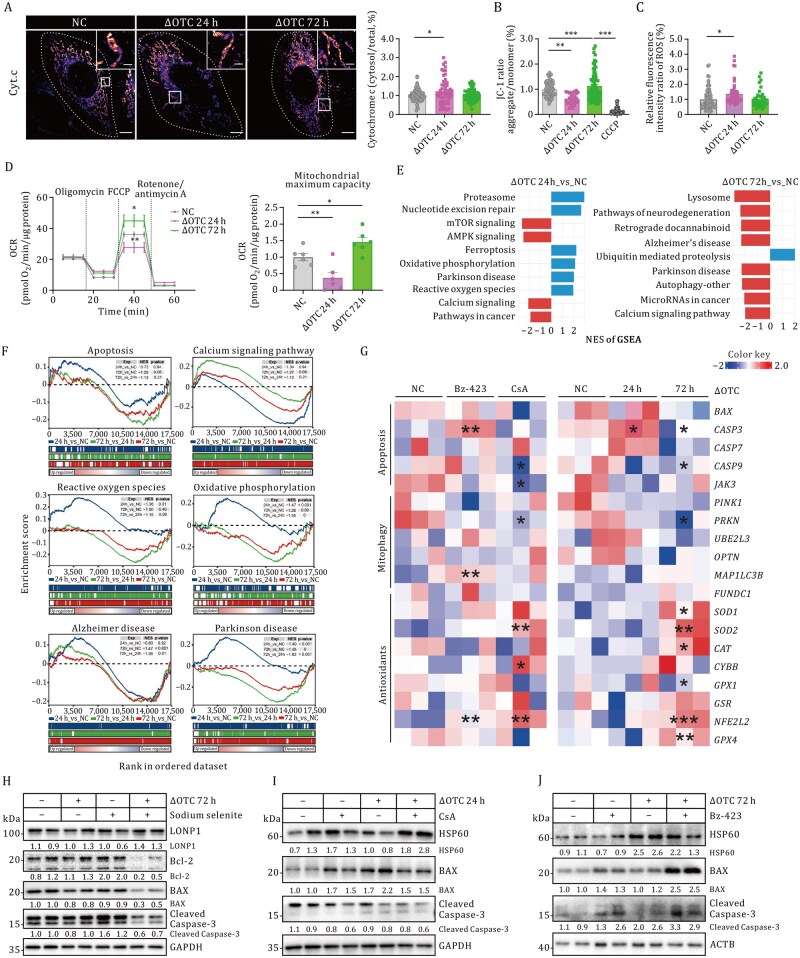
**Mitochondrial calcium fluxes regulate cell survival through UPR^mt^ activation**. (A) Immunolocalization of cytochrome c in untreated and ΔOTC-transfected U2OS cells visualized by GI-SIM. Scale bars, 5 µm (main images); 1 µm (insets). (B) Ratiometric ­detection of JC-1 fluorescence intensity (J-monomer/J-aggregates ± SEM). (C) Quantification of MitoSOX fluorescence intensity (means ± SEM). (D) Oxygen consumption rate (OCR) was measured over time after sequential addition of mitochondrial function modulators. Maximal respiratory capacity is shown. (E and F) Gene Set Enrichment Analysis (GSEA) results visualized as bar plots (E) and enrichment curves (F). (G) RNA-seq heatmap of the apoptotic pathway across mitochondrial calcium modulation. Color scale: log_2_(fold-change) (blue, low; red, high). (H–J) Quantification of apoptotic protein expression under different conditions. Loading controls: GAPDH/ACTB. Statistical analyses were performed using multiple unpaired t-tests (*P < 0.05, **P < 0.01,***P < 0.001). See also Fig. S5.

To determine whether mitochondrial calcium dynamics mediate the cytoprotective effects of UPR^mt,^ we conducted functional phenotyping under pharmacological (CsA/Bz-423) and ΔOTC transfection (24 h/72 h)-induced calcium perturbation conditions. Functional assessment showed that enhanced MERCS improved mitochondrial resilience, as indicated by reduced cytochrome c release (ΔOTC 72 h: 32% reduction vs. ΔOTC 24 h, Fig. 5A), stabilized membrane potential (ΔΨm 1.8-fold higher, Fig. 5B) following 24 h ΔOTC challenge, decreased mitochondrial Reactive oxygen species (ROS) production (MitoSOX intensity: 1.3-fold decrease) (Fig. 5C) and increased bioenergetic capacity (augmenting maximal oxygen consumption rate by 35%, *P *< 0.05) (Fig. 5D). Signaling pathway analysis revealed phase-dependent adaptive responses: acute protein aggregation (24 h ΔOTC) activated PD-related pathways (NES = 2.1), while chronic stress (72 h ΔOTC) suppressed apoptosis-related networks (NES = –2.2, Fig. 5E). Complementary GSEA (Gene Set Enrichment Analysis) confirmed biphasic pathway modulation, with early ROS buffering (24 h ΔOTC) transitioning to late apoptotic silencing (72 h ΔOTC, Fig. 5F; Tables S4 and S5). Transcriptome analysis demonstrated coordinated downregulation of apoptosis effectors (*CASP3*: 3.2-fold downregulation; *BAX*: 1.2-fold) and mitophagy regulators (*PINK*1: 1.3-fold) in both 72 h ΔOTC (right panel) and CsA-treated groups (left panel), paralleled by significant upregulation of antioxidant defense genes (*SOD2*: 2.5-fold; *GPX1*: 2.7-fold) (Fig. 5G). The protective role of UPR^mt^ was further validated by attenuated STS (sodium selenite)-induced apoptosis, which was hindered upon decreased HSP60 expression (Figs. 5H, S5A, and S5B). Pharmacological dissection showed that suppressing mitochondrial calcium transients with CsA recapitulated UPR^mt^-mediated protection (Cleaved Caspase-3: 72% reduction in ΔOTC/CsA vs. control, Figs. 5I and S5C), an effect reversed by Bz-423 (Figs. 5J and S5D). Together, these results establish that mitochondrial calcium transients orchestrate UPR^mt^-dependent cell survival through transcriptional silencing of apoptotic executors and metabolic reprogramming for stress adaptation.

### Mitochondrial calcium transient remodeling sustains neuronal survival in Alzheimer’s disease

Mounting evidence implicates sustained mPTP opening as a pathogenic hub linking bioenergetic collapse to necrotic neurodegeneration (Jadiya et al., 2019; Valasani Koteswara Rao et al., 2014). To elucidate the neuroprotective role of nanoscale calcium transients in AD pathophysiology, we conducted comparative analyses of mitochondrial calcium extrusion kinetics between WT N2a neuroblastoma cells and APPswe-expressing N2a cells (a validated AD cellular model harboring the Swedish mutant amyloid precursor protein) (Thinakaran et al., 1996).

**Figure 6.**
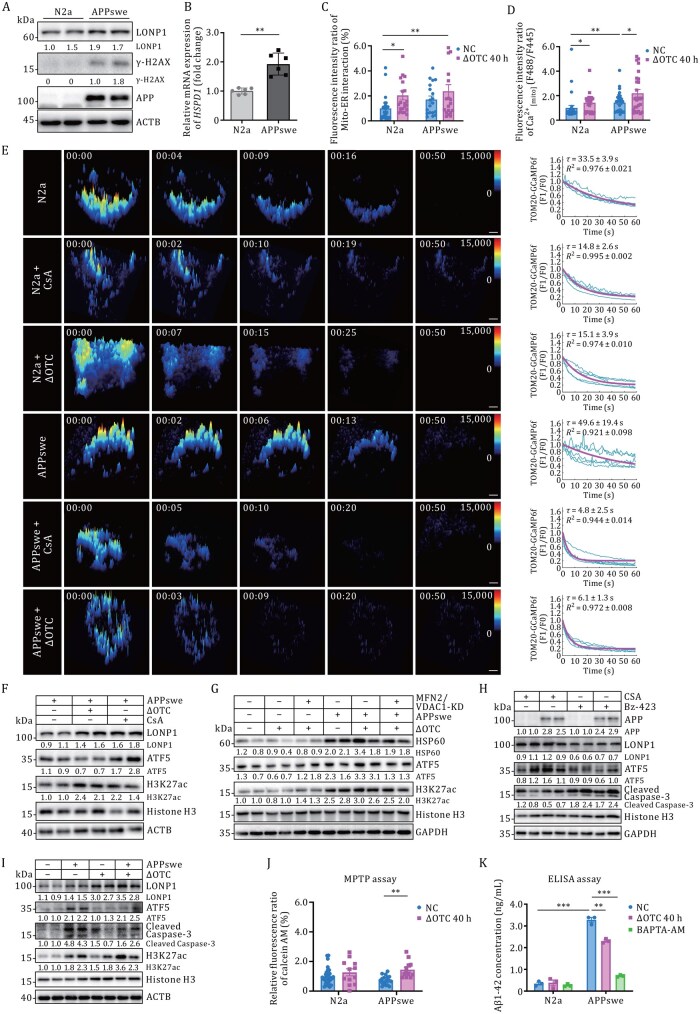
**UPR^mt^ alleviates APPswe cell apoptosis via inhibition of mitochondrial calcium extrusion**. (A) Western blot analysis showing exogenous transduction of the APPswe and Lonp1 expression in N2a and APPswe stable-expressing N2a (N2a-APPswe) cells. (B) Quantification of mRNA expression levels of HSP60 in N2a and APPswe cells. Statistical analysis was performed by two-tailed unpaired Student’s *t*-test compared to the N2a cell, **P < 0.01. (C) Quantitative analysis of the ratio of Mito-ER interaction revealed by Split-GFP recombination assay in the control and ΔOTC-transfected N2a or APPswe cells. ANOVA was used. *P < 0.05, **P < 0.01. (D) Statistical analysis of the fluorescence intensity ratio of mito-CFP-GCaMP6s. Comparison of means (±SEM) between ΔOTC-transfected and control cells in N2a and APPswe cells. ANOVA was used, *P < 0.05, **P < 0.01. (E) 3D time-series surface plots of traced Ca^2+^ transients detected by GI-SIM in N2a and APPswe cells. Representative fluorescent traces of periodic mitochondrial Ca^2+^ transients recorded by Tom20-GCaMP6f from five randomly selected cells in each group are shown as blue curves, and exponential fitting of the fluorescence decay is shown in magenta. Scale bars, 5 µm. (F and G) Quantification of UPR^mt^ marker protein levels (LONP11, ATF5, H3K27ac, HSP60) in control (E) or dual-siRNA (F) treated cells under ΔOTC transfection or CsA treatment. (H and I) Quantification of apoptotic protein levels. Protein levels were normalized to Actin, GAPDH, or Histone H3 and set to 1.00 in the corresponding control cells. (J) Calcein-quenching assay for mPTP opening. mPTP opening results in calcein release from mitochondria to cytosol, leading to reduced fluorescence. (K) ELISA measurements of Aβ1-42 levels in N2a and APPswe culture medium. Comparison of means (±SEM) between ΔOTC-transfected and control samples in N2a and APPswe cells. Two-way ANOVA was used. **P < 0.01, ***P < 0.001. See also Fig. S6.

Western blot analysis validated the elevated soluble APP derivatives and DNA damage marker γ-H2AX in APPswe cells (Fig. 6A), concomitant with moderate upregulation of mitochondrial quality control regulators Lonp1 and HSP60 compared with controls (Fig. 6A and 6B), suggesting engagement of the mitochondrial UPR^mt^. Split-GFP recombination experiments exhibited enhanced Mito-ER tethering in AD models (Split-GFP intensity: 1.2-fold increase vs. control) (Fig. 6C), confirmed by elevated mitochondrial calcium concentration under ΔOTC transfection (Figs. 6D and S6A).

Systematic quantification of calcium flux decay kinetics exposed prolonged mitochondrial Ca^2+^ oscillations in APPswe cells (APPswe: τ = 49.6 ± 19.4 s, *n *= 11; N2a: 33.5 s ± 3.9 s, *n *= 14). Pharmacological and genetic interventions—including ΔOTC transfection and CsA treatment—significantly accelerated Ca^2+^ clearance, mimicking the effect of mPTP inhibition (N2a + CsA: τ = 14.8 ± 2.6 s, *n *= 12; APPswe + CsA: τ = 4.8 ± 2.5 s, *n *= 20; N2a + ΔOTC: τ = 15.1 ± 3.9 s, *n *= 16; APPswe + ΔOTC: τ = 6.1 ± 1.3 s, *n *= 17; Fig. 6E; Video S6).

Western blot analysis confirmed robust UPR^mt^ activation upon treatment with ΔOTC, CsA, or CypD-KD in both control and AD models (Figs. 6F, S6B and S6H). This activation was abolished by either disrupting Mito-ER tethering (Figs. 6G and S6J), inhibiting histone acetylation with C646 (Fig. S6C), or promoting mPTP opening with Bz-423 (Figs. 6H and S6K). These conserved responses across U2OS and neuronal models establish calcium homeostasis modulation as a critical rheostat coupling mitochondrial proteostatic responses with AD-associated degenerative cascades.

Functional rescue assays demonstrated that pharmacological abrogation of mitochondrial calcium transients (ΔOTC/CsA) exerted neuroprotection through apoptosis suppression (reduction in Cleaved Caspase-3, Figs. 6H, 6I, S6K and S6L; attenuated Terminal deoxynucleotidyl transferase dUTP Nick End Labeling assay (TUNEL) intensity in ΔOTC, CypD-KD, or CsA group, Fig. S6D and S6F) and improved mitochondrial homeostasis restoration (mPTP closure efficiency elevated: 1.9-fold vs. control, Fig. 6J; Aβ1-42 clearance potentiation: 1.4/4.4-fold increase vs. control, Figs. 6K, S6E and S6G). The results indicate that the compromised mitochondrial calcium efflux serves as a cytoprotective role in Aβ1-42-induced neuronal apoptosis and mitigates Aβ1-42 toxicity (Fig. S6H). This multi-modal protection establishes nanodomain mitochondrial calcium modulation as a therapeutic strategy to decouple mPTP-driven neurodegeneration in AD.

## Discussion

### MERCS restructure mitochondrial calcium flux to orchestrate adaptive stress signaling

Mito-ER interactions serve as critical signaling hubs coordinating intracellular Ca^2+^ homeostasis, bioenergetic regulation, and apoptosis (Bravo-Sagua et al., 2017; Chen et al., 2017; Monaco et al., 2015). While sustained mPTP activation is known to dissipate mitochondrial membrane potential (ΔΨ_mt_), impair oxidative phosphorylation, and trigger cell death (Bernardi et al., 2023). Yet the regulatory interplay between MAMs plasticity, mitochondrial calcium flux, and mitochondrial stress response pathways remains mechanistically undefined. Notably, the potential protective role of MAM-mediated UPR^mt^ activation in modulating mitochondrial efflux kinetics during proteotoxic stress constitutes an understudied area of organelle crosstalk.

Our experimental findings establish that ΔOTC-induced proteotoxic stress initiates a MFN1/2-dependent reinforcement of MAMs connectivity (Fig. 1), which spatially constrains mitochondrial calcium transients. GI-SIM demonstrated that mitochondrial calcium extrusion kinetics are mechanistically coupled to MAMs remodeling during UPR^mt^ activation (Figs. 2D and 3A), with complete abolition of this phenomenon observed in both MFN2-KO and cells subjected to MAM-disrupting interventions. Key findings demonstrate that ΔOTC-induced proteostatic stress triggers MFN2-dependent MAMs expansion (Fig. 2D), which gates mitochondrial calcium efflux and subsequent UPR^mt^ initiation (Fig. 3).

While our findings establish MERCS structural dynamics as critical modulators of compartmented mitochondrial Ca^2+^ handling, fundamental mechanistic gaps persist regarding the nanoscale molecular architecture underlying this regulation. Key unresolved questions include whether MERCS expansion restructures Ca^2+^ flux at organelle contact sites, particularly through spatial reorganization of IP3R/VDAC clusters, or localized regulation of MCU/EMRE complexes requires systematic investigation. Future studies could explore whether leucine zipper/EF hand-containing transmembrane-1 (LETM1) or mitochondrial import machinery fine-tunes calcium nanodomains during stress. Furthermore, the potential involvement of lipid transfer proteins (e.g., VPS13D, GRAMD1C) in coordinating membrane curvature with calcium channel positioning at MERCS presents an essential avenue for future mechanistic studies. To discriminate among these possibilities, multidisciplinary approaches combining optogenetic or pharmacologic modulation of ΔΨm, calcium channel, lipidomic profiling of contact sites, and real-time imaging in genetically engineered models will be crucial. Ultimately, integrating these perspectives will provide a more comprehensive understanding of how MERCS achieve precise spatiotemporal control over Ca^2+^ signaling in health and disease.

### MERCS orchestrate UPR^mt^ via combined calcium-related chromatin remodeling and ATF5 shuttling

UPR^mt^ activation is regulated at the epigenetic and transcriptional levels. Here, we identify that enhanced MAMs connectivity attenuates mitochondrial Ca^2+^ efflux, thereby triggering dual regulatory axes: (i) Transcriptional licensing through ATF5 nuclear translocation, and (ii) Epigenetic reprogramming via CAMK4-mediated H3K27ac deposition, that governing UPR^mt^ activation (Fig. 4). Intriguingly, MFN2-KO cells exhibited defective ATF5 nuclear accumulation—a phenotype rescued by cyclosporine A (CsA) treatment (Fig. 4B)—suggesting that Mito-ER tethering modulates mitochondrial outer membrane calcium transients, a prerequisite for ATF5 import. Immunofluorescence analysis further indicated that UPR^mt^ activation influences ATF5 trafficking, potentially through regulation of the TOM/TIM mitochondrial import machinery (Fig. S4A), a mechanism warranting further investigation.

Mounting evidence indicates that mitochondrial stress induces specific chromatin reorganization to facilitate UPR^mt^ activation (Merkwirth et al., 2016; van de Ven et al., 2017). Using 3D-SIM and Lattice light-sheet microscopy, we confirmed the chromatin structure changes caused by mitochondrial unfolded protein accumulation, and chromatin decondensation was positively correlated with UPR^mt^ activation (Figs. 4K, 4L and S4C), as previously reported (Li et al., 2021), prompting us to select H3K27ac as a marker of UPR^mt^. Notably, UPR^mt^-suppressed cells exhibited reduced H3K27ac deposition—a defect reversible upon CsA or BAPTA-AM treatment (Fig. 4H)—placing impaired calcium efflux downstream of MAMs in the regulation of this epigenetic modification. The inverse correlation observed between nuclear reticulum (NR) foci and H3K27ac levels (Fig. S4C) is consistent with reported competition between stress-responsive and differentiation-related chromatin states (Bootman et al., 2009; Galiova et al., 2008).

Given calcium’s emerging role as an epigenetic modulator (Hernandez-Oliveras and Zarain-Herzberg, 2024; Nair et al., 2006; Wang et al., 2016), we further investigated the underlying axis using CAMK4 gain- and loss-of-function approaches. Our results establish CAMK4 as a critical mitochondrial calcium-sensitive mediator linking mitochondrial stress to phase-specific chromatin remodeling (Fig. 4M–P). Although our data indicate that CAMK4 mediates calcium-dependent chromatin changes via CREB phosphorylation (Figs. 4O and S4H), the precise spatiotemporal mechanisms governing CAMK4 activation require further elucidation.

### Targeting mitochondrial calcium transients restores neuronal proteostasis: a novel therapeutic axis for Alzheimer’s disease

Previous studies suggest that the inhibition of mPTP activation using both pharmacological (cyclosporine-A and its derivatives) and genetic means (CypD*-*KD) reduces neuronal dysfunction and degeneration in both cell culture and mutant mouse AD models (Du et al., 2008, 2011; Valasani Koteswara Rao et al., 2014). Enhancement of the UPR^mt^ is cytoprotective in stressed primary mouse chondrocytes and cardiac against apoptosis (Smyrnias et al., 2019; Zhou et al., 2022b), UPR^mt^ markers are also involved in cellular stress protection, including increased levels of LONP1 inhibiting apoptosis and enhancing cell survival under hydrogen peroxide (H_2_O_2_), hypoxia, and ultra-violet stresses (Cheng et al., 2013); HSP60 plays a novel role in mitochondrial permeability transition, contributing to a cytoprotective chaperone network that antagonizes CypD (encoded by *PPIF*)-dependent cell death in tumors (Ghosh et al., 2010). However, the role of the UPR^mt^-mediated nanodomain calcium homeostasis in disease progression has not been extensively studied.

**Figure 7.**
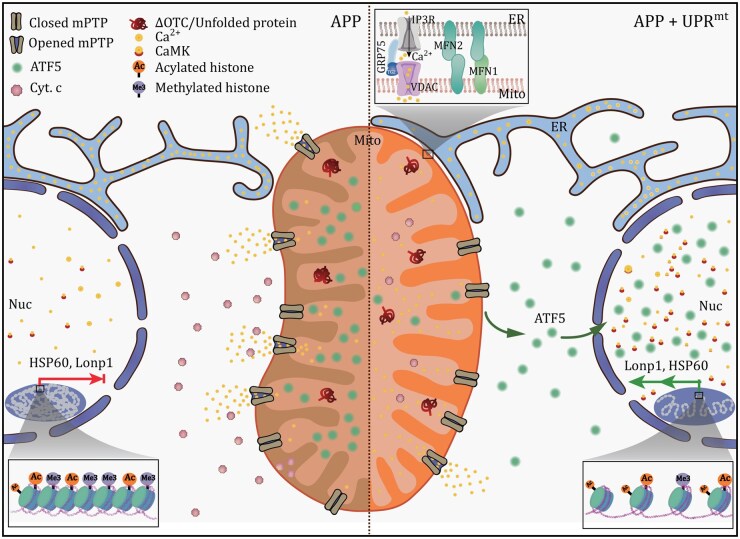
**Working model for calcium-mediated UPR^mt^ activation**. Schematic illustration showing the role of Ca^2+^ transients in the protection of AD cells against apoptosis.

In this study, we examined how calcium transients regulate UPR^mt^ activation and AD progression. We found that UPR^mt^ was more strongly activated by CsA or CypD-KD than by VBIT-4—a compound reported to protect against AD-related neuronal loss in mice (Belosludtsev et al., 2024; Verma et al., 2022) but with minimal effect on mPTP opening (Xian et al., 2022). All Aβ1‑42-induced apoptotic effects were reversed by either CsA treatment or ΔOTC transfection (Figs. 6 and S3). These results indicate that enhancing Mito-ER contacts or attenuating mitochondrial calcium efflux alleviates mitochondrial dysfunction and suppresses apoptosis in AD neurons by sustaining UPR^mt^ activation through mitochondrial calcium retention (Fig. 7).

Notably, our work provides mechanistic evidence that mitochondrial calcium transients mechanistically contribute to both UPR^mt^ activation and mitigation of amyloid-beta pathology in AD models. The upregulation of both ATF4 and ATF5 in UPR^mt^-insufficient APPswe cells (data not shown) suggests that AD pathogenesis may involve additional factors beyond UPR^mt^—such as ER stress—or that UPR^mt^ engages multiple signaling axes governed by distinct transcription factors. Nevertheless, sustained UPR^mt^ activation through modulation of Mito-ER contacts or mitochondrial calcium efflux represents a rational and potential therapeutic strategy for AD (Fig. 7).

### Limitations of the study

Our study has several limitations. It remains largely unknown how unfolded protein aggregation induces Mito-ER tethering, which impedes mPTP closing rather than NCLX, and subsequent attenuated Ca^2+^ transients on the mitochondrial outer surface, and how these attenuated mitochondrial Ca^2+^ transients trigger ATF5 translocation from mitochondria to nucleus to activate UPR^mt^. Additionally, due to chromatin reorganization contributing to UPR^mt^ activation, further studies are needed to elucidate the relationship between calcium-sensitive catalysts and chromatin remodeling.

It is possible that other Ca^2+^ channels (e.g., LETM1, etc.) or mitochondrial import machinery may be involved in reducing long-lasting, high-amplitude, or highly localized mitochondria Ca^2+^ transients, but these are not detected by our GI-SIM analysis of the change of TOM20-GCaMP6f fluorescence intensity. The role of sufficient UPR^mt^ initiation induced by CsA or interfering Mito-ER interactions in the AD mouse model should be explored in the future. Nevertheless, our study provides insights into the regulatory mechanisms of organelle interactions in UPR^mt^ activation in mammalian organisms.

## Supplementary Material

pwaf109_Supplementary_Data

## Data Availability

Not applicable.

## Code availability

All software used in this study is publicly available.
