# Supplementary material for: Spatiotemporal Ca2+ nanodomain remodeling at MERCS regulates mitochondrial proteostasis
Source: Protein Cell. 2025 Dec 8;17(4):304–19. doi: 10.1093/procel/pwaf109 (PMC13107556; doi:10.1093/procel/pwaf109)
Supplement: pwaf109_Supplementary_Data [file pwaf109_supplementary_data.zip › PAC-25443-GYT-Supplemental materials-Methods.pdf]

METHOD DETAILS

Plasmids

A model for the accumulation of protein aggregates within the mitochondrial matrix was constructed by making a mutant form of the mitochondrial matrix protein, ornithine transcarbamylase (OTC), by deletion of amino acids 30-114, encompassing the carbamyl phosphate-binding domain (ΔOTC)(Zhao et al., 2002). ΔOTC-mCherry and ΔOTC-Halo were constructed by inserting the truncated OTC into pmCherry-N1 and Halo-N vectors, respectively. Human cDNA encoding *ATF5* was inserted into mEmerald-C1 to construct ATF5-mEmerald (deposited at Addgene under ID 248207). To construct TOM20-GCaMP6f probe facing the cytosol, GCaMP6f was fused to the C terminus of the *TOMM20* transmembrane domain (deposited at Addgene under ID 248205). A mitochondrial matrix targeting peptide was subcloned into the N terminus of CFP-linker-GCaMP6s vector to construct a ratiometric mitochondrial Ca^{2+} sensor (mito-CFP-GCaMP6s) as previously reported(Li et al., 2020)to detect mitochondrial calcium concentration.

Cell culture and gene transfection

U2OS cells were cultured in McCoy's 5A medium supplemented with 10% FBS at 37°C with 5 % CO_2 as previously described. N2A and APPswe cells were grown in DMEM medium supplemented with 10% FBS at 37°C with 5 % CO_2, and APPswe cells were kept selected by addition of 1 mg/mL G418. We transiently transfect cells with lipo3000 as the instruction manual.

Living-cell imaging

Cells were plated on collagen-coated high-NA coverslips (Gibco, #A1048301), and mitochondria in U2OS and neuroblastoma cell lines were transfected with plasmids 18-24 hours prior to imaging or pre-incubated with dyes. High temporal-spatial resolution images were captured by custom-built GI-SIM(Guo et al., 2018) and 3D-SIM under the physiological conditions of 37°C and 5% CO_2, and images were acquired for live cells at excitation NA of 1.35. GI-SIM achieves an imaging speed of up to 684 Hz for 60,000 frames. Labview (version 2019) were used for image acquisition.

Lattice light-sheet images of U2OS cells were acquired on the Lattice Lightsheet 7 imaging system (Zeiss, Germany) with a 13.3×/0.44 NA Excitation water objective and a 44.83×/1.0 NA detection water objective (Zeiss), using solid-state lasers (488 nm, 561 nm, 640 nm) and two sCMOS (Complementary Metal-Oxide-Semiconductor) cameras (Hamamatsu ORCA-Fusion). The length of light plane is 30 μm， and the thickness of the beam is 1,000 nm. Lattice light-sheet image stacks were processed by de-skewing

and deconvolution method. The x-y-z axis resolution was 290 nm × 290 nm × 330 nm.

REDMAP system

The REDMAP system(Zhou et al., 2022) is a red/far-red light-mediated, reversible protein binding system based on the plant photoreceptor PhyA, which rapidly binds to the shuttle protein far-red elongated hypocotyl 1 (FHY1) under illumination with 660-nm light with dissociation occurring at 730 nm. To enhance the interaction between Mitochondria and ER, the light-induced binding characteristics of phyA and FHY1 proteins are primarily exploited. TOM20 and calnexin, Mito and ER marker proteins, are fused to PhyA and FHY1, respectively, to facilitate this interaction. The modified plasmids (1 μg each) were co-transfected into cells. Subsequently, the cells were exposed to LED red light for 1 minute and then proceeding with subsequent imaging experiments or Western blotting experiments.

Split-GFP recombination experiment

We constructed TOM20-FP1-10 and Calneixin-FP11×7 plasmid facing the cytosol, as previously reported(Pham et al., 2024, Cabantous et al., 2005). The reassembly of two fragments leads to a fluorescent readout, which is widely used to monitor protein-protein interactions.

OCR assay

To detect mitochondrial respiration, the oxygen consumption rate (OCR) was recorded when cells were metabolically perturbed by the sequential injections of oligomycin (1 μM, final concentration), carbonyl cyanide p-(trifluoromethoxy) phenylhydrazone (FCCP) (1μM, final concentration), and rotenone/antimycin A (0.5 μM, final concentration). Mitochondrial respiration in intact cells were recorded using the Oroboros O2k.

Resting $Ca^{2+}_{[mito]}$ Measurements

U2OS cells were grown on coverslip and transfected with ratiometric mitochondrial-targeted Ca^{2+} sensor plasmids, together with the plasmids of interest, to test resting $Ca^{2+}_{[mito]}$ with high sensitivity. After 24 or 72 hours, imaging of cell containing dishes loaded with HBSS was performed on a homemade GI-SIM microscope equipped with a 100×/1.49 NA objective. Cells were alternatively illuminated at 560 nm, 488 nm and 445 nm and fluorescence was collected through a 647LP, 515/30 nm and 561/50 nm band-pass filter (Semrock). The exposure time was set to 20 ms at 488 nm and 445 nm. The averaged 488/445 ratio (F488/445) was used as the mitochondrial free Ca^{2+} concentration. Analysis was performed with ImageJ(Patron et al., 2014). Images were background- corrected frame by frame by subtracting the mean pixel values of a cell-free region of interest.

Dynamic $Ca^{2+}_{[mito]}$ measurement and analysis

Cells were plated on collagen coated coverslips and transfected with the mitochondrial-targeted Ca^{2+} sensors (TOM20-GCaMP6f, LaminB1-GCaMP6f or mito-GCaMP6s) along with plasmids of interest for various time. Imaging was performed using a 100×/1.49 NA oil objective (Olympus), laser beams of 488 nm (500 mW, Coherent, Genesis-MX-SLM), 560 nm (500 mW, MPB Communications, 2RU-VFL-P-500-560), 642 nm (500 mW, MPB Communications, 2RU-VFL-P-500-642) and a sCMOS camera (Photometrics Kinetix) on GI-SIM. These cells are briefly incubated with appropriate Halo-Tag ligands (Promega, #GA1110, #GA1121) or SNAP substrate (NEB, #S9102S) which readily crosses the cell membrane, before imaging. The live-cell imaging setup was stable and well controlled in order to maintain the cells at 37 °C and 5% CO_2 for imaging. To acquire transient calcium signals, lower laser power and shorter exposure time were used to minimize photobleaching. The exposure time for each image was 10 ms, at a 1-second interval for 120 seconds. The TOM20-GCaMP6f, mito-GCaMP6s and LaminB1-GCaMP6f signals were analyzed for 120 seconds during the following periods as indicated in the figures: 20 minutes after RCI incubation (2 μM Ryanodine, 10 μM Anisomycin, and 250 μM 2-Aminoethyl diphenylborinate); 2 min immediately after 100 μM histamine addition; 15 mins after addition of 50 mM BAPTA-AM (#AB120503); or 120 min after addition of 5 μM CsA (MCE, #HY-B0579). For analyzing Ca^{2+} amplitude, the baseline fluorescence (F0) is subtracted from the peak fluorescence (F), and the transient amplitude was calculated as ΔF/F0 = (F-F0)/F0. Global mitochondrial and nuclear Ca^{2+} transients were counted.

Definition of τ

To mitigate human selection bias across different treatment conditions, we randomly selected morphologically normal cells for fluorescence signal recording. Each cell was continuously imaged for 60 seconds. The overall fluorescence intensity of each image was obtained by extracting the calcium signal intensities at mitochondrial locations across all frames. All fluorescence intensities were normalized to the first image captured at t = 0. The data presented are from three independent experiments conducted under blinded conditions.

After obtaining the time-dependent calcium signal trajectories, we fitted the entire 60-second dataset using an exponential function to calculate the fluorescence decay lifetime (τ). To minimize the influence of outliers, τ values were derived from individual exponential fits for each of the five cells under each treatment condition and then averaged to enhance the reliability of the results. The fitting equation used was as follows:

$$I(t) = I_0 * e^{-t/\tau} + I_{bg}$$

where $I(t)$ represents the fluorescence intensity at time t, I_0 denotes the initial fluorescence intensity of GCaMP6 at $t = 0$, τ is the fluorescence lifetime (i.e., the time required for the intensity to decay to 1/e of its initial value), and I_{bg} represents the background fluorescence signal.

In the results analysis, for clearer visualization of the overall data trend, we first averaged the five calcium signal intensity-time curves within each group to obtain a representative trend curve. This averaged curve was then fitted using the exponential function described above, and the resulting fitted curve is depicted in purple in the figures to intuitively illustrate the dynamic characteristics of fluorescence decay.

Determination of Ca^{2+} Affinity (Kd)

We recorded baseline fluorescence from cells expressing GCaMP6f or GCaMP6s in culture medium (10 min, frames every 5–10 s). F_{min} was measured after a 5-min perfusion with a Ca^{2+}-free, EGTA-containing solution by averaging images from minutes 5–7. Likewise, F_{max} was determined after a 5-min perfusion with a solution containing saturating $CaCl_2$, using the same imaging window. For calibration, we applied intracellular solutions with defined free [Ca^{2+}] (40 nM to 50 mM) in ascending order. Each concentration was equilibrated for 4–5 min, followed by two images taken 1 min apart; the mean fluorescence (F) at each step was recorded. Background-corrected fluorescence was normalized as $F_{norm} = (F - F_{min}) / (F_{max} - F_{min})$. The resulting F_{norm} values were plotted against free [Ca^{2+}] and fitted to the Hill equation to derive the apparent Kd and Hill coefficient (n).

Isolation of nuclear, mitochondrion and cytoplasmic fractions

Cells were transfected with the mCherry-ΔOTC for varied time (24h and 72h). The transfected cells were washed three times with phosphate-buffered saline (PBS), detached from the culture flask with trypsin, and centrifuged at 500g for 10 min. Mitochondria were freshly isolated using the mitochondria isolation kit as manufacturer's instructions (QIAGEN, #37612). Similarly, cytoplasmic and nuclear fractions were separated using a Nuclear and Cytoplasmic Protein Extraction Kit (Beyotime, #P0028) according to manufacturer's recommended procedures. The protein concentration in the supernatant was quantified using BCA protein assay kit (Beyotime, #P0010) and then subjected to immunoblotting as described previously.

Western blot analysis

Western blot analysis was performed according to standard methods. Briefly, total protein was extracted using RIPA buffer (Beyotime, #P0013B) supplemented with PMSF (protease inhibitor) and phosphatase inhibitor cocktail to protects proteins from degradation by endogenous proteases and phosphatases. After being quantified by a BCA assay (Beyotime, #P0010), protein samples were separated by SDS-PAGE and transferred onto PVDF membranes. The PVDF membranes were incubated with primary and secondary antibodies at the indicated dilutions. The primary antibodies used in this study were as follows: rabbit anti-ATF4 (1:2500, CST, #11815S), rabbit anti-ATF5 (1:2000, Abcam, #ab184923), rabbit anti-LONP1 (1:2000, Proteintech, #16512-1-AP), rabbit anti-H3K27ac (1:2000, Abcam, #ab4729), rabbit anti- H3K9me3 (1:2000, Abcam, #ab8898), rabbit anti-HSP60 (1:2500, Abcam, #ab190828). Mouse

anti-GAPDH antibody (1:10000, Abcam, ab8245) and rabbit anti-β-actin antibody (1:1000, CST, #8457) were used as internal controls, and an HRP-conjugated goat anti-rabbit/mouse antibody was used as the secondary antibody. Protein bands were detected using the ECL method. The density of immunoblot bands of the images was quantified using Image J software (NIH Image). All western blot assays in our study were repeated at least three times with consistent results.

Immunostaining and TUNEL staining

Cells cultured on coverslips were fixed with 4% PFA for 20 min, and then permeabilized with 0.2% Triton for 15 min at room temperature. After blocking with 5% goat serum for 1 h, cells were incubated with the indicated primary antibodies (diluted in 5% goat serum) overnight at 4 °C. After washing 3 times with PBS, cells were incubated with fluorophore-conjugated secondary antibodies for 1 h at room temperature. Samples were imaged using GI-SIM microscope equipped with a 1.49NA oil-immersion objective lens. TUNEL staining was performed using a TUNEL Assay Kit (Beyotime, #C1086) according to the manufacturer's protocol.

MitoSOX analysis

Sample cells were stained with MitoSOX Red (Thermo Fisher, M36005). A 1 mM stock solution of MitoSOX Red reagent was prepared by dissolving the contents of the vial in 10 μL of DMF. To prepare the working solution, 10 μL of the 1 mM stock solution was added to 10 mL of HBSS containing calcium and magnesium. The working solution was applied to cover cells adhering to coverslips in a 35 mm dish. Cells were incubated for 30 minutes at 37°C and 5% CO_2. Cells were gently washed three times with warm buffer (HBSS containing calcium and magnesium) and imaged immediately after washing.

JC-1 assay

Cells were plated on collagen-coated coverslips and transfected with Halo-ΔOTC for various durations. For analysis of mitochondrial membrane potential, sample cells and positive control cells (treated with carbonyl cyanide 3-chlorophenylhydrazone [CCCP]) were stained with the JC-1 fluorescent probe (10 μg/mL, Beyotime, #C2005) at 37°C, 5% CO_2 for 20 min. JC-1 fluorescence was measured following excitation at 488 nm and 560 nm, with emission signals for monomers and aggregates detected using 561/50 nm band-pass and 647 nm long-pass filters (Semrock), respectively.

mPTP assay

The mitochondrial permeability transition pore (mPTP) opening was detected using an mPTP Fluorescence Assay Kit (Beyotime, #C2009S) according to the manufacturer's instructions. Briefly, cells were washed twice with prewarmed PBS and treated with Calcein AM staining solution. Cells were incubated at 37 °C for 30 min in the dark,

followed by replacement of the staining solution with prewarmed culture medium containing 10% FBS for another 30 min incubation at 37°C in the dark. Samples were imaged using a custom-built GI-SIM microscope equipped with a 1.49NA oil-immersion objective lens within 10 minutes.

ChIP-qPCR analysis

Chromatin Immunoprecipitation (ChIP) assay was performed using the ab500 ChIP kit (Abcam, #ab500) according to the manufacturer's instructions. Briefly, U2OS cells were cross-linked with formaldehyde (final concentration 1.1%) and buffer A, followed by quenching with glycine (125 mM). The cells were washed in ice-cold PBS and lysed using the lysis buffer provided in the kit. Chromatin was sheared by sonication (15 min; 15 s ON/OFF) to obtain chromatin fragments between 200 bp and 1000 bp. Histone-bound DNA was immunoprecipitated with specific antibodies: Histone H3 (Proteintech, #17168-1-AP), H3K27ac (Abcam, #AB4729), and H3K9me3 (Abcam, #AB8898) overnight at 4 °C. The sonicated chromatin extract was also incubated with immunoglobulin G (IgG) antibody (Cell Signaling Technology, #2729S) as a negative control. The antibody/chromatin samples were incubated with Protein A magnetic beads. After washing, DNA was purified and 200 ng was used for qPCR using 2 × ChamQ Universal SYBR qPCR Master Mix (Vazyme, #Q711-03-AA) in the CFX96 Real-Time PCR Detection System.

The qPCR signals were derived from the input sample taken early during the ChIP procedure. After the ChIP procedure, qPCR was used to amplify and quantify the DNA fragments immunoprecipitated with specific antibodies. The percentage of input DNA that was immunoprecipitated was calculated by dividing the qPCR signal from the ChIP sample by the qPCR signal from the input sample and multiplying by 100. The resulting percentage represents the relative enrichment of the specific DNA sequence in the ChIP sample compared to the total DNA present before immunoprecipitation.

RNA-seq analysis

RNA sequencing was performed on samples from the negative control (NC), ΔOTC 72h, and ΔOTC 24h groups. Differentially expressed genes were identified using the limma package in R (version 4.4.0), with significance thresholds set at $|\log2(\text{fold change})| > 1$ and adjusted $p < 0.05$. Gene Set Enrichment Analysis (GSEA) was conducted using the clusterProfiler package to identify enriched biological pathways. Results were visualized using bar plots and enrichment curves generated with ggplot2 and GseaVis in R.

Real-time qPCR

Total RNA was isolated from cells using TRIzol Reagent (ambion, #15596026). Reverse transcription was performed on 1 µg of total RNA from each sample using the

PrimeScript™ RT reagent Kit with gDNA Eraser (Takara, #RR047A) in a total volume of 20 µL, according to the supplier's instructions. The reverse-transcribed cDNAs were stored at -40°C until qPCR. For real-time analysis, cDNA was diluted 20-fold. Quantitative real-time PCR was performed using 2 × ChamQ Universal SYBR qPCR Master Mix (Vazyme, #Q711-03-AA) in the CFX96 Real-Time PCR Detection System. The relative mRNA expression was normalized to β-actin and quantified using the ΔΔCT method.

Statistical analysis

All experiments were repeated at least three times. For immunofluorescence staining, cells or images were randomly selected for analysis. Graphs and p-values were generated using GraphPad Prism 8.0 software. Frequency distributions and nonlinear fitting were performed using Origin2024 software. The density of immunoblot bands and immunofluorescence intensity were quantified using ImageJ software (NIH Image). No statistical methods were used to predetermine sample size or to assess whether the data met the assumptions of the statistical tests used. Statistical comparisons were made using two-tailed unpaired Student's t-tests, one-way ANOVA, and two-way ANOVA, with results shown as mean values ± SEM. Significance levels are indicated as follows: ns, no significant difference; *p < 0.05; **p < 0.01; ***p < 0.001.

Image rendering

Three-dimensional (3D) rendering was performed using Imaris software (Bitplane, AG). The Imaris Surfaces tool was used to create 3D renderings of each channel based on local fluorescence intensities. Two-dimensional (2D) rendering was performed using Fiji software(Schindelin et al., 2012).

Aβ1-42 sandwich Elisa tests

Concentrations of human Aβ1-42 were measured in total protein extracts from control and ΔOTC-transfected groups using ELISA kits (MIbio, #ml027504-48T) according to the manufacturer's instructions. Optical densities of standards and samples were measured at 450 nm using a microplate reader. Aβ1-42 levels in samples were normalized to total protein levels and expressed as pg/mL.

References

CABANTOUS, S., PEDELACQ, J. D., MARK, B. L., NARANJO, C., TERWILLIGER, T. C. & WALDO, G. S. 2005. Recent advances in GFP folding reporter and split-GFP solubility reporter technologies. Application to improving the folding and solubility of recalcitrant proteins from Mycobacterium tuberculosis. *J Struct Funct Genomics,* **6**, 113–9.

GUO, Y., LI, D., ZHANG, S., YANG, Y., LIU, J. J., WANG, X., LIU, C., MILKIE, D.

E., MOORE, R. P., TULU, U. S., KIEHART, D. P., HU, J., LIPPINCOTT-SCHWARTZ, J., BETZIG, E. & LI, D. 2018. Visualizing Intracellular Organelle and Cytoskeletal Interactions at Nanoscale Resolution on Millisecond Timescales. *Cell,* 175**,** 1430–1442 e17.

LI, J., WANG, L., CHEN, Y., YANG, Y., LIU, J., LIU, K., LEE, Y. T., HE, N., ZHOU, Y. & WANG, Y. 2020. Visible light excited ratiometric-GECIs for long-term in-cellulo monitoring of calcium signals. *Cell Calcium,* 87**,** 102165.

PATRON, M., CHECCHETTO, V., RAFFAELLO, A., TEARDO, E., VECELLIO REANE, D., MANTOAN, M., GRANATIERO, V., SZABO, I., DE STEFANI, D. & RIZZUTO, R. 2014. MICU1 and MICU2 finely tune the mitochondrial Ca2+ uniporter by exerting opposite effects on MCU activity. *Mol Cell,* 53**,** 726–37.

PHAM, T. D., POLETTI, C., TIENTCHEU, T. M. N., CUCCIOLONI, M., SPURIO, R., FABBRETTI, A., MILON, P. & GIULIODORI, A. M. 2024. FAST, a method based on split-GFP for the detection in solution of proteins synthesized in cell-free expression systems. *Sci Rep,* 14**,** 8042.

SCHINDELIN, J., ARGANDA-CARRERAS, I., FRISE, E., KAYNIG, V., LONGAIR, M., PIETZSCH, T., PREIBISCH, S., RUEDEN, C., SAALFELD, S., SCHMID, B., TINEVEZ, J. Y., WHITE, D. J., HARTENSTEIN, V., ELICEIRI, K., TOMANCAK, P. & CARDONA, A. 2012. Fiji: an open-source platform for biological-image analysis. *Nat Methods,* 9**,** 676–82.

ZHAO, Q., WANG, J., LEVICHKIN, I. V., STASINOPOULOS, S., RYAN, M. T. & HOOGENRAAD, N. J. 2002. A mitochondrial specific stress response in mammalian cells. *EMBO J,* 21**,** 4411–9.

ZHOU, Y., KONG, D., WANG, X., YU, G., WU, X., GUAN, N., WEBER, W. & YE, H. 2022. A small and highly sensitive red/far-red optogenetic switch for applications in mammals. *Nat Biotechnol,* 40**,** 262–272.

Supplemental Figures

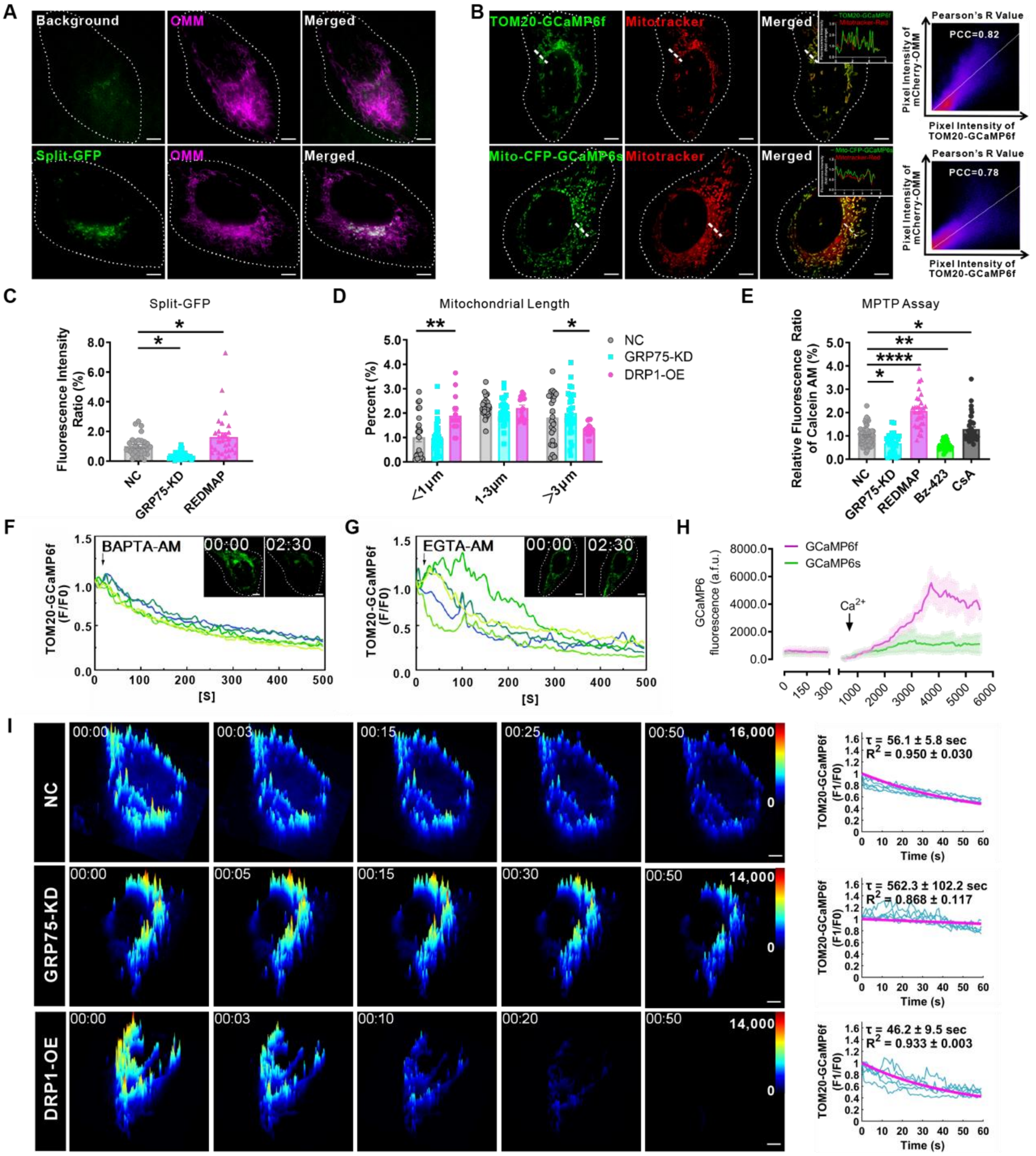

Figure S1. Enhanced MAMs specifically constrain mitochondrial calcium transients, related to Figure 1. (A) Representative SIM images of the Split-GFP-based contact site labeling system. (B) Quantification of sublocations of mito-CFP-GCaMP6f or TOM20-GCaMP6f in live cells. (C) Relative fluorescence intensity of Split-GFP in GRP75-KD or REDMAP-treated cells compared to control. (D) Mitochondrial length in GRP75-KD or DRP1-OE groups relative to control. (E) mPTP opening assessed by calcein-quenching assay. Data were analyzed by One-way ANOVA; *$p < 0.05$, **$p < 0.01$, ****$p < 0.0001$. (F-G) Traces of mitochondrial surface Ca^{2+} transients following BAPTA-AM or EGTA-AM treatment. (H) Calibration of Ca^{2+}-sensing properties for mitochondrial-targeted GCaMP6f and GCaMP6s. mito-CFP-GCaMP6s exhibited higher Ca^{2+} affinity (Kd = 141 ± 7 nM) compared to TOM20-GCaMP6f (Kd = 356 ± 48 nM). Hill coefficients were 2.5 ± 1.3 for GCaMP6s and 5.50 ± 2.74 for GCaMP6f (n = 5, mean ± SEM). (I) 3D time-series surface plots of

Ca^{2+} transients captured by GI-SIM in GRP75-KD or DRP1-OE groups versus control. Representative fluorescence traces (blue) from five randomly selected cells per group across three independent experiments are shown, with exponential decay fits in magenta. Scale bars: 5 μm (A-B, I).

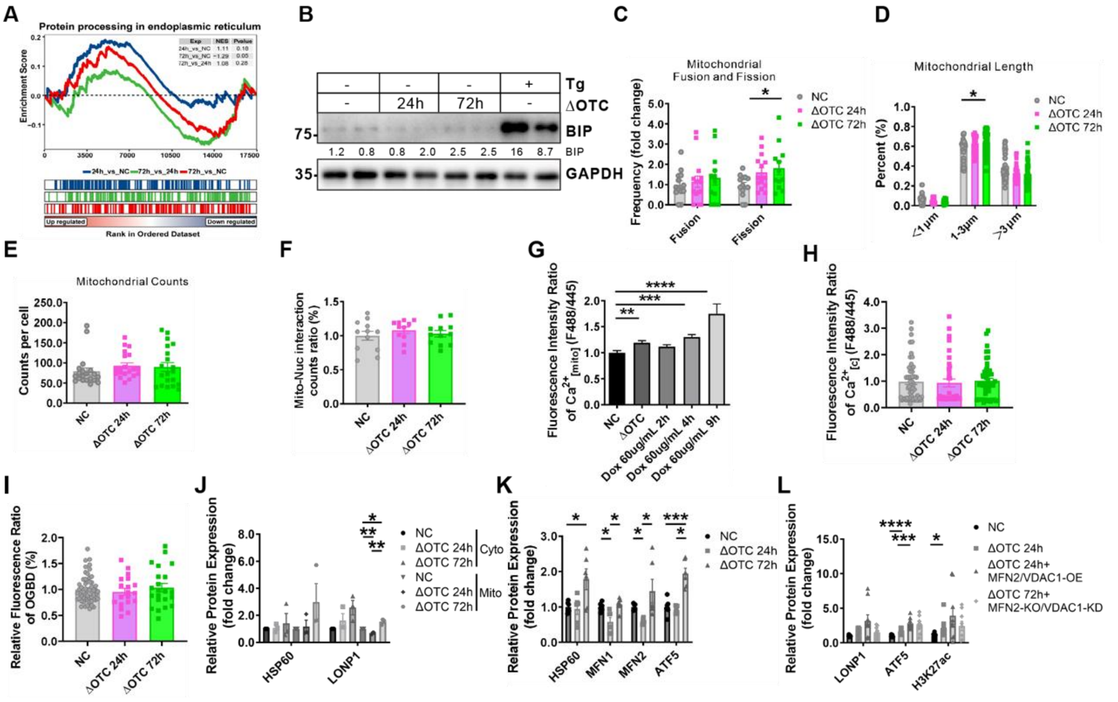

Figure S2. Enhanced Mitochondria-ER interactions promote UPR^{mt} activation, related to Figure 2. (A) Gene set enrichment analysis (GSEA) enrichment curves for ER stress pathways. (B) Western blot analysis of the ER stress marker BIP in short- or long-term ΔOTC-expressing cells, with thapsigargin (Tg) and untreated cells as positive and negative controls, respectively. (C-F) Quantification of mitochondrial dynamics in ΔOTC-expressing cells versus control. (G) Fluorescence intensity ratios of mito-CFP-GCaMP6f in HeLa cells under ΔOTC or doxycycline time-course treatment. (H) Relative cytosolic Ca^{2+} levels in control and ΔOTC-expressing Hela cells. (I) Lysosomal Ca^{2+} levels in control and ΔOTC-expressing cells. Data analyzed by unpaired two-sided t-test versus untreated controls. (J-K) Protein expression levels of UPR^{mt} markers under indicated conditions. Protein levels were normalized to β-actin (ACTB) or Histone H3, with control set to 1.00. Two-way ANOVA was used for statistical comparison. *$p < 0.05$, **$p < 0.01$, ***$p < 0.001$, ****$p < 0.0001$.

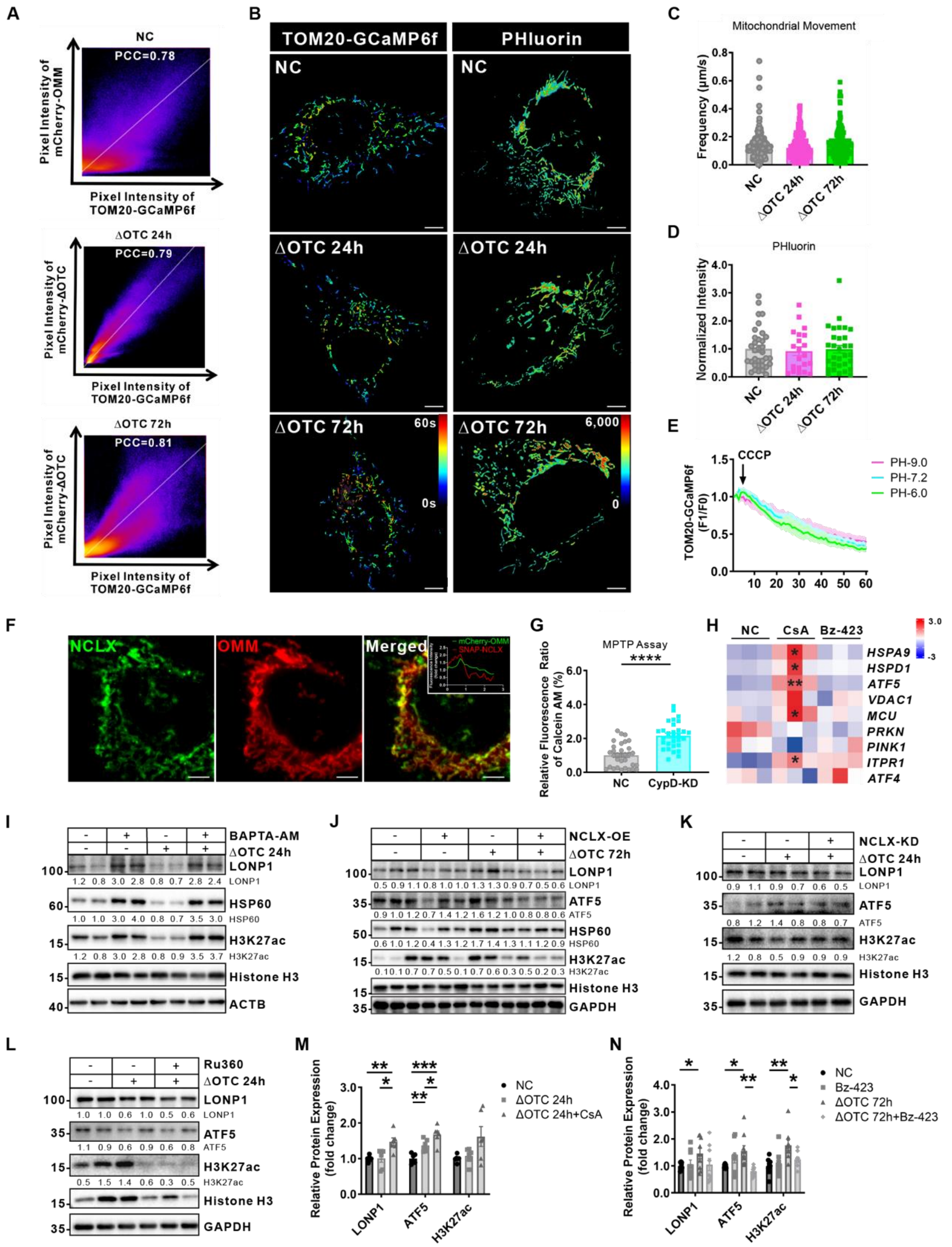

Figure S3. Inhibition of mPTP Opening Constrains Mitochondrial Calcium Transients, Related to Figure 3. (A) Quantitative colocalization analysis demonstrating stable mitochondrial localization of TOM20-GCaMP6f in ΔOTC-expressing versus control cells, indicated by high Pearson's correlation coefficients (PCC). (B-D) Quantification of mitochondrial movement and outer membrane pH in ΔOTC-expressing cells compared to control. (E) Trace of Ca^{2+} on the mitochondria surface under CCCP treatment in buffers of different pH. (F) Representative images of NCLX-Snap subcellular localization. Scale bars: 5 μm (B, F). (G) mPTP opening assessed by calcein-quenching

assay. Unpaired two-sided t-test; ****p < 0.0001. (H) Clustered heatmap showing expression of UPR^{mt}-related genes. Color scale represents $\log_2$ fold change (blue: low, red: high). (I-N) Protein levels of UPR^{mt} markers under BAPTA-AM (I), NCLX-OE (J), NCLX-KD (K), and Ru360 (L) treatments relative to untreated controls. Protein expression was normalized to ACTB, GAPDH, or Histone H3, with control set to 1.00. Two-way ANOVA; *p < 0.05, **p < 0.01.

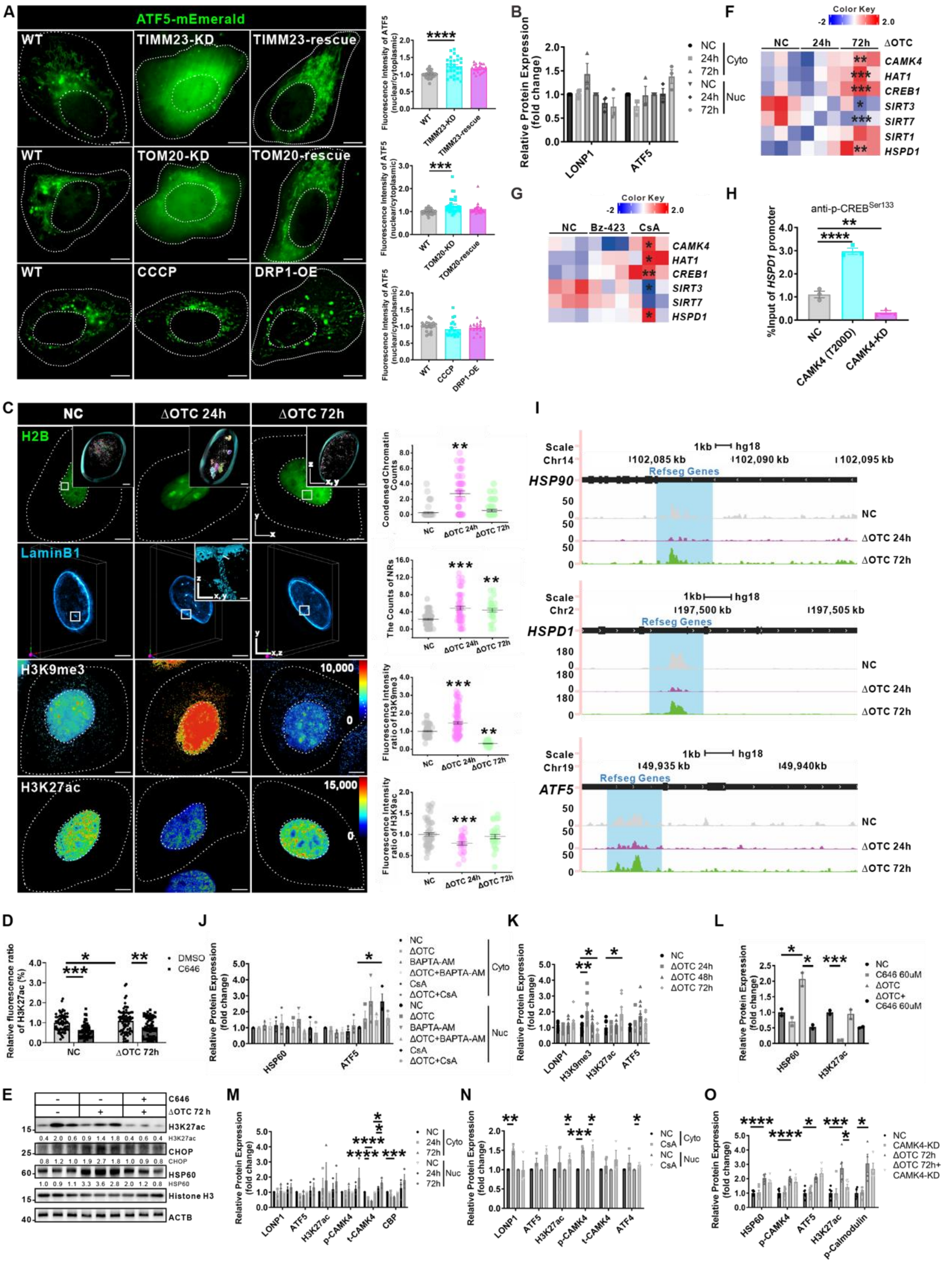

Figure S4. Calcium-dependent chromatin remodeling axis in UPR^{mt} activation, related to Figure 4.

(A) Subcellular redistribution of ATF5-mEmerald in WT cells versus those with TIMM23 or

TOM20 perturbation (knockdown or rescue). Scale bars: 5 µm; quantification results are shown accordingly. (B) Nuclear-to-cytoplasmic (N/C) ratio of ATF5 from subcellular fractionation assays. (C) Representative SIM images and quantitative analysis of chromatin organization and H3K27ac modification under early (24 h) and sustained (72 h) ΔOTC-induced mitochondrial stress. 3D chromatin reconstructions (Imaris) are inset. Unpaired two-sided t-test; $^{**}p < 0.01$, $^{***}p < 0.001$. Scale bars: 5 µm (2D), 2 µm (3D). (D) Immunofluorescence intensity of H3K27ac in control and ΔOTC-expressing cells, with or without C646 treatment. Two-way ANOVA; $^{*}p < 0.05$, $^{**}p < 0.01$, $^{***}p < 0.001$. (E) UPR^{mt} marker expression in 72 h ΔOTC-expressing cells treated with C646. Protein levels normalized to ACTB or Histone H3; control set to 1.00. (F, G) RNA-seq heatmaps of calcium-responsive epigenetic regulators (*CAMKK/CAMK4/HAT1*) under ΔOTC expression (F) or CsA/Bz-423 treatment (G). Color scale: $\log_2$ fold-change (blue: low; red: high). One-way ANOVA; $^{*}p < 0.05$, $^{**}p < 0.01$, $^{***}p < 0.001$. (H) ChIP-qPCR analysis of p-CREB enrichment at the *HSPD1* promoter in cells expressing constitutively active CAMK4 (T200D) or following CAMK4-KD. Data normalized to input; one-way ANOVA; $^{*}p < 0.05$, $^{**}p < 0.01$, $^{***}p < 0.001$, $^{****}p < 0.0001$. (I) ATAC-seq profiles of H3K27ac enrichment at *HSPD1, HSP90,* and *ATF5* regulatory regions (blue background) in untreated, 24 h ΔOTC (magenta), and 72 h ΔOTC (green) conditions. (J–O) Protein levels of UPR^{mt} markers (J), H3K27ac (K, L), and p-CAMK4 (M–O) under genetic or pharmacological perturbations. All values normalized to ACTB, GAPDH, or Histone H3; control set to 1.00. Two-way ANOVA; $^{*}p < 0.05$, $^{**}p < 0.01$, $^{***}p < 0.001$, $^{****}p < 0.0001$.

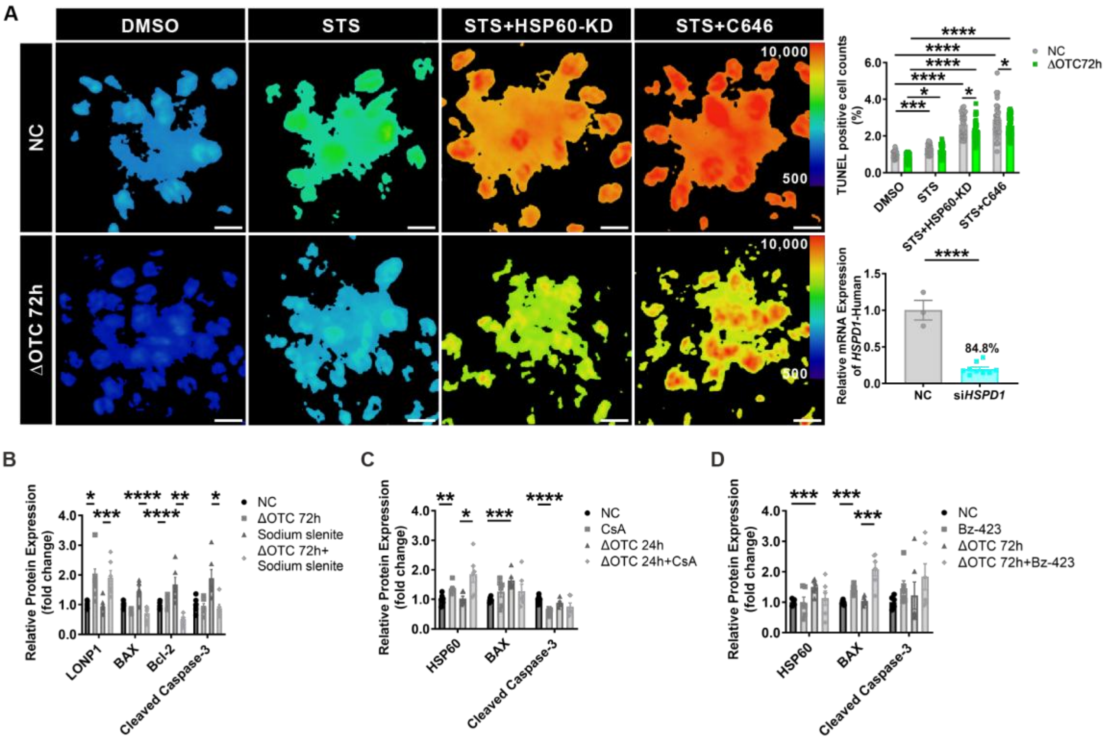

Figure S5. UPR^{mt} activation promotes cell survival, related to Figure 5. (A) Quantification of apoptotic cells by TUNEL assay. The ratio of TUNEL-positive cells per field is shown (right), with representative pseudo-colored images (left) indicating fluorescence intensity (blue: low; red: high). Scale bar: 15 µm. Data are presented as mean ± SEM. (B-D) Protein levels of apoptosis markers

following genetic or pharmacological induction of UPR^{mt}. All values were normalized to ACTB, GAPDH, or Histone H3, with control set to 1.00. Two-way ANOVA; *p < 0.05, **p < 0.01, ***p < 0.001, ****p < 0.0001.

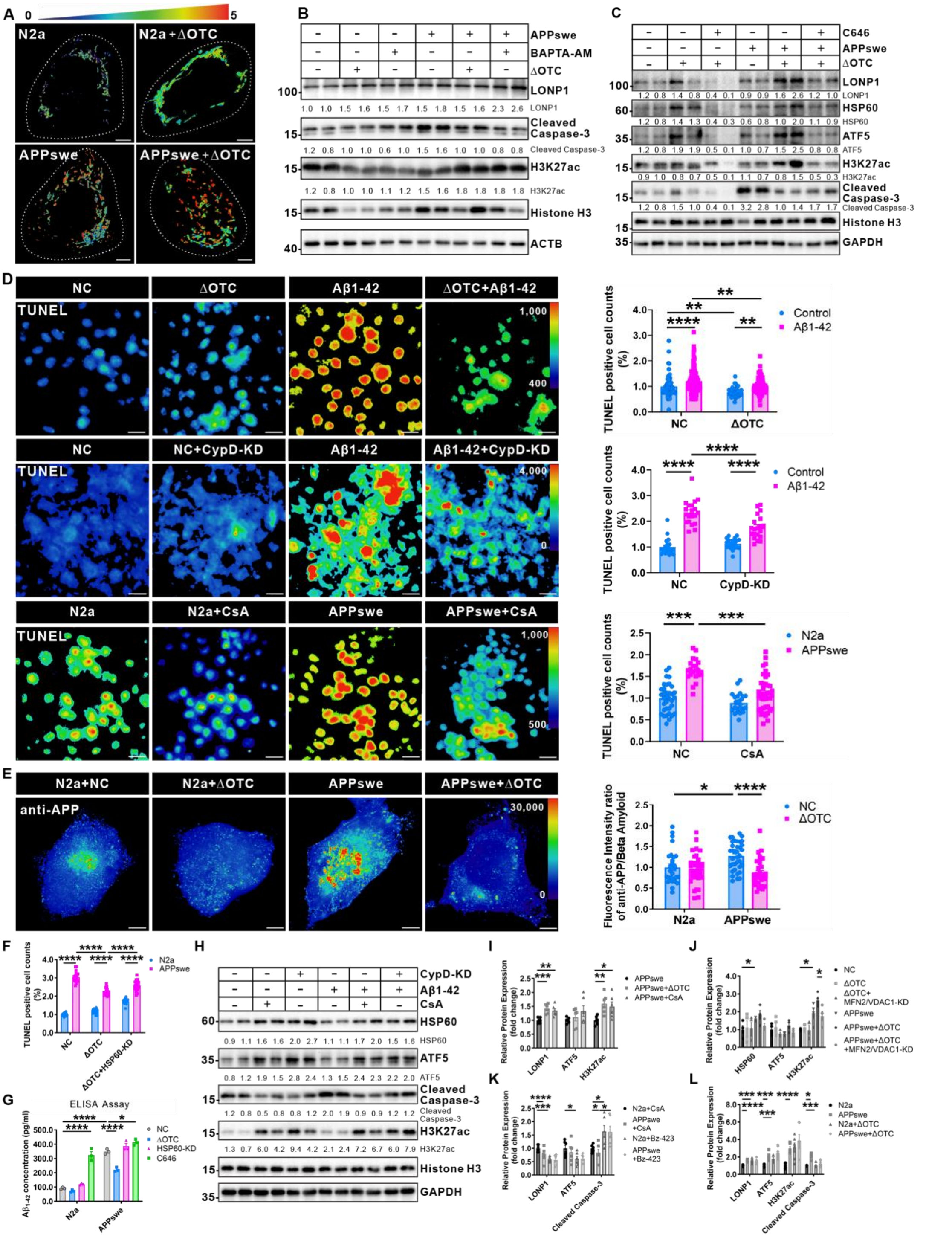

Figure S6. Constrained mitochondrial calcium-induced UPR^{mt} activation protect against apoptosis, related to Figure 6. (A) Representative ratiometric images (F488/F445) of mito-CFP-GCaMP6s, pseudo-colored to indicate calcium levels. Scale bar: 5 μm. (B–C, H) Apoptotic protein expression in N2a and APPswe cells under UPR^{mt}-inducing conditions (B: BAPTA-AM or ΔOTC; C: C646 treatment; H: CsA or CypD-KD). Protein levels normalized to β-actin or Histone H3; control set to

1.00. (D, F) Quantitative analysis of apoptotic cell death by TUNEL assay. The fluorescence ratio of apoptotic cells compared to control cells per microscopic field of view was determined and graphically represented. Pseudo-color bars indicate the intensity of green fluorescence in representative fields (blue, low; red, high). Scale bar: 15 μm. (E) typical cellular images showing immunostaining for intracellular APP oligomers. (G) ELISA measurement of secreted Aβ1–42 in culture medium from N2a and APPswe cells under indicated treatments. (I-L) Protein levels of H3K27ac (I-J) and apoptosis markers (K-L) following genetic or pharmacological treatment as indicated. All values normalized to ACTB, GAPDH, or Histone H3; control set to 1.00. Two-way ANOVA; $^{*}p < 0.05$, $^{**}p < 0.01$, $^{***}p < 0.001$, $^{****}p < 0.0001$.

Supplemental Tables

Table 1. Overview of ATAC-seq Raw Data Quality Metrics

**Peak_ID**	**ΔOTC72h**	**NC**	**start**	**end**	**Region**	**Gene name**	**Description**
NC_000019.10_49929737_49930137	65.5187352714201	50.9363514752203	49929738	49930137	Promoter (<=1kb)	ATF5	cyclic AMP-dependent transcription factor ATF-5
NC_000002.12_197499783_197500183	235.711623512951	277.515294244304	197499784	197500183	Promoter (<=1kb)	HSPD1	60 kDa heat shock protein, mitochondrial
NC_000014.9_102086832_102087232	61.9654267997965	26.3463886940795	102086833	102087232	Promoter (<=1kb)	HSP90AA1	heat shock protein HSP 90-alpha isoform 1

**Peak_ID**	**ΔOTC24h**	**NC**	**start**	**end**	**Region**	**Gene name**	**Description**
NC_000019.10_49929737_49930137	47.3018951647475	43.5757619070402	49929738	49930137	Promoter (<=1kb)	ATF5	cyclic AMP-dependent transcription factor ATF-5
NC_000002.12_197499783_197500183	130.487986661372	237.412771769391	197499784	197500183	Promoter (<=1kb)	HSPD1	60 kDa heat shock protein, mitochondrial
NC_000014.9_102086832_102087232	19.5731979992059	22.5391871932967	102086833	102087232	Promoter (<=1kb)	HSP90AA1	heat shock protein HSP 90-alpha isoform 1

**Peak_ID**	**ΔOTC72h**	**ΔOTC24h**	**start**	**end**	**Region**	**Gene name**	**Description**
NC_000019.10_49929737_49930137	46.3250181955523	39.6131915699679	49929738	49930137	Promoter (<=1kb)	ATF5	cyclic AMP-dependent transcription factor ATF-5
NC_000002.12_197499783_197500183	167.716483486151	109.277769848187	197499784	197500183	Promoter (<=1kb)	HSPD1	60 kDa heat shock protein, mitochondrial
NC_000014.9_102139529_102139929	36.3097541567955	51.906940677889	102139530	102139929	Promoter (<=1kb)	HSP90AA1	heat shock protein HSP 90-alpha isoform 1
